# OTUs and ASVs Produce Comparable Taxonomic and Diversity from Shrimp Microbiota 16S Profiles Using Tailored Abundance Filters

**DOI:** 10.3390/genes12040564

**Published:** 2021-04-13

**Authors:** Rodrigo García-López, Fernanda Cornejo-Granados, Alonso A. Lopez-Zavala, Andrés Cota-Huízar, Rogerio R. Sotelo-Mundo, Bruno Gómez-Gil, Adrian Ochoa-Leyva

**Affiliations:** 1 Departamento de Microbiología Molecular, Instituto de Biotecnología (IBT), Universidad Nacional, Autónoma de México (UNAM) Avenida Universidad #2001, Colonia Chamilpa, Cuernavaca, Morelos 62210, Mexico; rodrigo.garcia@ibt.unam.mx (R.G.-L.); fer.cornejog@gmail.com (F.C.-G.); 2 Departamento de Ciencias Químico Biológicas, Universidad de Sonora (UNISON), Blvd., Rosales y Luis, Encinas, Hermosillo, Sonora 83000, Mexico; alexis.lopez@unison.mx; 3 Camarones el Renacimiento S.P.R. de R.I. Justino Rubio 26, Colonia Ejidal, Higuera de Zaragoza, Sinaloa 81330, Mexico; el_andres_cota@hotmail.com; 4 Laboratorio de Estructura Biomolecular, Centro de Investigación en Alimentación y Desarrollo, A.C. Hermosillo, Sonora 83304, Mexico; rrs@ciad.mx; 5 Centro de Investigación en Alimentación y Desarrollo, A.C. Mazatlán, Sinaloa 82100, Mexico; bruno@ciad.mx

**Keywords:** shrimp microbiota, 16S profiling, OTUs, ASVs, clustering methods, denoising

## Abstract

The interplay between shrimp immune system, its environment, and microbiota contributes to the organism’s homeostasis and optimal production. The metagenomic composition is typically studied using 16S rDNA profiling by clustering amplicon sequences into operational taxonomic units (OTUs) and, more recently, amplicon sequence variants (ASVs). Establish the compatibility of the taxonomy, α, and β diversity described by both methods is necessary to compare past and future shrimp microbiota studies. Here, we used identical sequences to survey the V3 16S hypervariable-region using 97% and 99% OTUs and ASVs to assess the hepatopancreas and intestine microbiota of *L. vannamei* from two ponds under standardized rearing conditions. We found that applying filters to retain clusters >0.1% of the total abundance per sample enabled a consistent taxonomy comparison while preserving >94% of the total reads. The three sets turned comparable at the family level, whereas the 97% identity OTU set produced divergent genus and species profiles. Interestingly, the detection of organ and pond variations was robust to the clustering method’s choice, producing comparable α and β-diversity profiles. For comparisons on shrimp microbiota between past and future studies, we strongly recommend that ASVs be compared at the family level to 97% identity OTUs or use 99% identity OTUs, both using tailored frequency filters.

## 1. Introduction

Crustacean production is one of the fastest-growing economic activities to embrace aquaculture as its primary source for commercial produce [1], with over 9.38 million tons of specimens produced worldwide as of 2018, accounting for 22% of the international aquatic species market. Since the Pacific whiteleg shrimp, *L. vannamei*, is the most commonly cultured shrimp species worldwide [2], several studies have explored its genetics and, more recently, the impact of bacteria in its digestive tract both under wild-type and standardized environmental condition in farms [3,4,5,6,7,8]. 

Organ or niche-specific bacteria have shown to contribute to the modulation of the shrimp’s immune response, its overall nutrient absorption, vitamin production, and the physiological development and regulation of its metabolic processes, which ultimately have a relevant impact on shrimp production and can be finely tuned in farms [5,6,7,9,10].

Thousands of different microorganisms populate the gastrointestinal tract of shrimp. However, the hepatopancreas and the intestine are widely different ecological niches. Each tissue presents specific environment-associated biochemical conditions and nutrient availability, and they are colonized by different sets of bacteria [4]. The taxonomic variations in the microbiota have been mainly explored using 16S rDNA profiling from environmental and aquaculture samples [4,6,7], frequently selecting up to two consecutive hypervariable regions [9,11]. A previous study determined that the hypervariable region V3 is a cost-effective alternative to determine the microbiota diversity in the shrimp hepatopancreas and the intestine [12]. Although amplicons spanning both the V3 and V4 regions bore a higher taxonomic resolution and diversity, the V3 region showed optimal family-level resolution and better performance than V4 at the genus level using current sequencing technologies [12].

By far, the most common type of clustering method for shrimp studies has been identity clustering, producing operational taxonomic units (OTUs), which use a fixed sequence identity cutoff, usually 97% for sequences from the same species [13]. However, different regions vary in their discriminating power in practice, and some phylogenetically-related taxa share a less predictable identity percentage [14]. 

In recent years, denoising has been introduced through several different popular algorithms as an alternative clustering method based on predicting and correcting actual sequencing errors (noise) before forming clusters, here referred to as amplicon sequence variants (ASVs) [15,16,17]. Thus, ASV clustering is based on sequence probability rather than sequence identity (as in OTUs). The denoising approaches use well-established statistical models to determine which low-prevalence sequences appear more than would be expected for artifacts and are therefore valid sequence variants. This results in fewer but self-consistent custom clusters that have been thoroughly validated to have a more acceptable precision [18,19,20]. In recent years, the field of microbiota research has been using ASVs increasingly often, in addition to the traditional OTUs methods. The differences of both identity and denoising methods have been explored on mock communities, soil, mouse feces, human milk, and intestinal samples [19,20,21,22,23,24] but remain largely unexplored in shrimp-related ecological niches.

To this date, few studies have used denoising methods to assess the microbiota in culturable aquatic species, and their obtained taxonomy, α, and β-diversity profiles need to be compared to OTUs findings to unify the microbiota discoveries. The first such study in *L. vannamei* was carried out for the microbiota of Malaysian and Vietnamese specimens by Zoqratt et al. in 2018 [25] focused on V3-V4 amplicons for studying differences in the *Vibrio* genus using a sequence-specific approach.

Now, the objectives of our study were: (i) to obtain adequate sequence filters that make the taxonomy comparable between OTUs and ASVs 16S profiling for shrimp microbiota, and (ii) to validate those sequence filters to identify the variations in the microbiota from different biological (organ) and environmental (pond) niches between OTUs and ASVs. To this end, we analyzed the 16S profiles from the shrimp intestine and hepatopancreas from different ponds using the same set of V3 amplicons with 97% and 99% identity OTUs ASVs using standard algorithms for 16S profiling [15,26]. Our study determined whether traditional and new clustering approaches are comparable in taxonomy, α, and β-diversity profiles for shrimp microbiota.

## 2. Materials and Methods

### 2.1. Sample Collection

**Figure 1:**
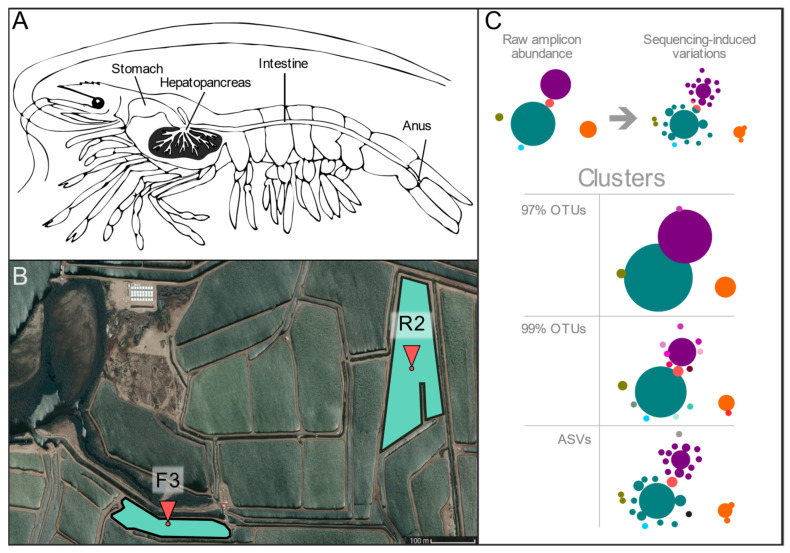
Pond location and anatomic distribution of shrimp organs. (**A**) Anatomical representation of L.vannamei studied organs, hepatopancreas, and intestine (both organs were taken from the same specimen). (**B**) Satellite overview of sample collection sites in Sinaloa, México (CNES/Airbus©, 2020). Six specimens were taken from each pond: F3 (26°01’45.4” N 109°23’52.9” W) and R2 (26°01’55.8” N 109°23’12.4” W). (**C**). Diagram showing the source of artefactual variation due to high-throughput sequencing and different types of clusters used in the present study. Circles represent abundance and colors different sequence identities.

Twelve cultured shrimp, identified as adult *L. vannamei* specimens by morphological keys [27], were obtained from two different farming ponds (water salinity ~40 ppm and temperature ~29 °C) in a farm from Northwest Pacific in Sinaloa, Mexico in summer 2016: Six from pond F3 (henceforth “E” samples, with coordinates 26°01’45.4” N 109°23’52.9” W) and six from pond R2 (“L” samples, at 26°01’55.8” N 109°23’12.4” W), shown in Figure 1. Shrimp were fed two times per day using commercial feed (~35% protein) for three months. The hepatopancreas and intestine were aseptically dissected in situ from each specimen and stored in an RNA-later solution at −80 °C until used. In total, 12 hepatopancreas and 12 intestines were collected for this study.

### 2.2. DNA Extraction, 16S rDNA Amplicon Preparation, and Sequencing

Total DNA was extracted from each individual organ with Quick-DNA Fecal/Soil Microbe Miniprep kit (Zymo Research, Irvine, CA, USA) following the manufacturer’s recommendations. The DNA integrity and concentration were then determined by Agarose gel electrophoresis and Qubit (LifeTechnologies, Carlsbad, CA, USA), respectively. Primers 338F (5’-ACTCCTACGGGAGGCAGCAG-3’) and 533R (5’-TTACCGCGGCTGCTGGCAC-3’, that have been broadly used for studying shrimp microbiota were used for amplifying the V3 hypervariable region of the 16S rDNA [28]; primer target sequences were selected so that the resulting PCR products were purified with AMPure XP beads (Beckman Coulter, Inc., Brea, CA, USA) and barcoded according to the sequencing library preparation user’s guide (Illumina, San Diego, CA, USA). Finally, the concentration was assessed with a Qubit fluorometer and the size distribution with an Agilent 2100 Bioanalyzer (Agilent Technologies, Santa Clara, CA, USA). The V3 libraries were sequenced in an Illumina MiniSeq platform (Illumina, San Diego, CA, USA) with as 2x150 Paired-Ends at the Research Center on Food and Development A.C. (CIAD) in Mazatlán, Sinaloa, Mexico.

### 2.3. Data Preprocessing

Two operational taxonomic units (OTU) sets were created with identity clustering methods, and one amplicon sequence variants (ASV) set was created with denoising methods to evaluate different clustering approaches for the V3 amplicon reads (Supplementary Figure S2.1). To achieve this, Illumina adapter sequences and amplification primers were previously removed with Cutadapt v2.0 [29]. For creating datasets 01_OTU_97 and 02_OTU_99, PRINSEQ v0.20.4 [30] was used to filter low entropy sequences, trim low quality 3’ and 5’-ends, and remove sequences with low mean quality reads were then joined with COPE v1.2.5 [31]. For creating the 03_ASV set, the Cutadapt-trimmed set was filtered to remove error-prone paired-end sequences with DADA2 v1.12 suite in R v3.6.0 [15,32]. More detailed methods are available in Supplementary File S1.

### 2.4. OTU Clustering (Identity-Based)

The COPE-joined set was clustered into operational taxonomic units (OTUs) with VSEARCH v2.7.0 [33] algorithm in QIIME2 v2019.1 [26] specifying open-reference clusters at 97% identity for set 01_OTU_97 and 99% identity for set 02_OTU_99 against Greengenes 13_5 references [34]. The QIIME 2 suite was selected for convenience and ease of use. The clustering algorithm was chosen as it uses a similar centroid approach to that of an earlier version of UCLUST, the default clustering approach in earlier versions of QIIME 1, one of the most common pipelines used for aquatic species’ 16S profiles. The 99% threshold was used to emulate the theoretical maximum identity achieved by DADA2.

### 2.5. ASV Clustering (Denoising)

The DADA2-filtered reads were used for constructing ASVs with DADA2 in R. This algorithm was selected due to its ease of use, open-access availability, and because it has no sequence-length limitations. Error models were created with all sequences and a maximum of ten iterations, followed by sample-independent denoising. Paired-End reads were then joined according to the V3 region overlap and filtering the resulting length. This produced set 03_ASV.

### 2.6. Chimera Filtering, Taxonomic Identification, and Filters

All downstream procedures were the same for all three sets. Sample-singletons were removed. Chimeras were detected with VSEARCH using the overlap of reference-based chimeras (Broad Institute gold database [35]) and *de novo* chimeras. Taxonomy identification was carried out with the 97% (set 01) and 99% (sets 02–03) Greengenes clusters as references, using the scikit-learn classifier v0.19.1 [36], and collated tables were created for all taxonomic levels. Features (OTUs, ASVs, or taxa) not comprising a minimum of 0.1% of the total abundance in any sample were removed using in-house R scripts to avoid filtering biases [37]. The reference database for taxonomy was selected mainly due to its ubiquity in the field, but the newer clusters available for QIIME2 were used to address some limitations and known bad assignations in the Pseudoalteromonadaceae and Vibrionaceae families were corrected beforehand.

### 2.7. Comparing the Performance of OTU and ASV Sets

In-house R scripts were used for evaluating differences in the actual sequences and the corresponding read composition of each cluster set. These sequence-based analyses were carried out with VSEARCH and explored the sequence overlaps between sets and how nucleotide variations accumulated, resulting in differential resilience of each set to clustering. Such information also provided an insight into the maximum cluster resolution of each method and the expected sequence redundancy in each set. Cluster/sequence tables were compared before and after frequency filters, considering total reads per set, total unique clusters/taxa, and their intersection (Supplementary Figure S2.2). Taxa having informative tags (those not having an empty terminal taxonomic node) were identified and used to calculate Spearman’s rank correlations between V3 sets. Differences between specific species were assessed between sets 01 and set 02-03. First, we analyzed taxa in sets 02-03 with <33% abundance of that reported in set 01 (i.e., predominant in set 01), and then, we analyzed taxa in sets 02–03 with ≥33% (i.e., predominant in sets 02–03).

### 2.8. α-Diversity Comparison (Within-Sample)

Diversity analyses were carried with Vegan (v2.5-6) [38] and in-house R scripts unless stated otherwise. Shannon entropy, total observed features (OUT/ASV/taxa), and Chao1 richness were estimated for each sample from 10,000 rarefied tables at a depth of the smallest sample. Shannon’s entropy is a proxy for feature diversity consisting of the calculation of predictability of each new draw that is taken from a random sample and is expressed as a natural logarithm. The higher the diversity, the greater the uncertainty of predicting the following items. The Chao 1 index is a calculation of the expected richness, consisting of the observed features plus additional uncaptured variation that is estimated by evaluating the ratio of low-frequency features (namely, singletons and doubletons). Samples were compared by Organ, Pond, and Organ-Pond using sample medians and an α = 0.05.

### 2.9. β-Diversity Comparison (Between-Sample)

Tables standardized with average rarefied observations were constructed to calculate Jaccard (absence/presence) and Bray–Curtis (abundance) dissimilarity matrices. Analysis of similarities (ANOSIM) tests was used to compare groups (as defined in the previous section) and carry out pairwise post hoc testing. Matrices were subjected to principal coordinate analysis (PCoA) ordinations. Standardized tables were used to construct weighted and unweighted UniFrac matrices based on phylogenetic reconstructions created with SEPP [39]. These distance matrices were analyzed as above.

## 3. Results

### 3.1. Different Preprocessing and Clustering Methods Produced Distinct Sets of Clusters

A total of 1,102,570 paired-end (PE) sequencing reads (mean = 45,940.42 ± 8766.43) were generated from the 24 biological samples, 12 hepatopancreas, and 12 complete intestines from the same specimens (Figure 1A), six from each of two ponds (Figure 1B). Sequences not flanked by the corresponding 16S primer sequences or having spurious Illumina adapters were removed, obtaining a total of 957,415 reads (39,892.29 ± 8592.20), which were used for ASVs and OTUs taxonomic profiling (Supplementary Table S3.1). The V3 sequences were subjected to different quality filters and sequence-joining procedures to create three types of sequence clusters, two from OTUs at 97 and 99% identity and one for ASVs, see Experimental Procedures (Figure 1C, Supplementary Figure S2.1). The most common types of pipelines for the creation of OTUs and ASV clusters are represented by 01_OTU_97 (mean = 34,202.00 ± 8088.06) and 03_ASV sets (mean 36,677.71 ± 7543.79), respectively, and set 02_OTU_99 (mean = 31,675.54 ± 7335.93) was created to compare the impact of using a higher clustering identity.

### 3.2. Sequence-Level Analyses Show Well-Outlined ASV Clusters and Partially Clusterable OTU Sets That Are Origin-Dependent

Following chimera filters and singleton removal, the set 03_ASV had the most sequences (854,841 reads), followed by 01_OTU_97 (780,472) and 02_OTU_99: (717,908). Notably, both OTU sets retained fewer total reads than the ASV set, hindered by singleton filters due to their vast number of low-abundance OTUs. These were clustered into 1407 ASVs and 4968 and 11,541 OTUs, respectively, each bearing one centroid (representative) sequence. The lower diagram shows the total reads that match these compartments (out of the total 2,353,221 in all three sets). Set 02_OTU_99 had the largest percentage of the complete read collection (96.58%), followed closely by the 03_ASV set (94.12%), and further by set 01_OTU_97 (91.48%).

**Figure 2:**
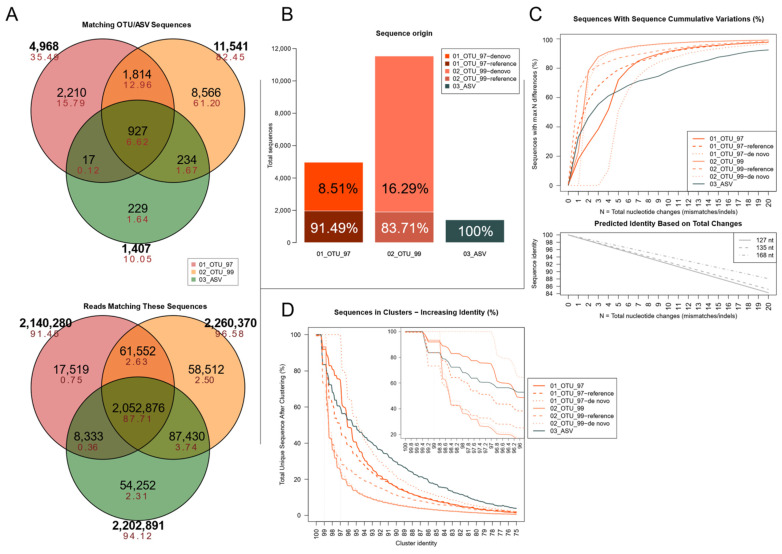
Sequence-level analyses showing methodological differences. (**A**) Venn diagram showing the centroid sequence overlap between the three methodological sets (top) and the corresponding total reads that match such sequences (bottom). Black = total sequences/reads; red = percentages. (**B**) Distribution of unique centroid sequences per set. Open-reference OTU sets include reference-based and *de novo* cluster sequences, presented as separate segments of each bar. Percentages of reads matching these sequences per set are shown for each segment. (**C**) Maximum mismatches between any pair of sequences in the sets. The x-axis (N) shows the total cumulative mismatches or indels allowed in each pairwise sequence alignment. The y-axis shows the percentage of all centroid sequences presenting a maximum of N punctual differences (how many of the centroid sequences differ in up to N total nucleotides). The graph below shows the expected sequence identity as changes accumulate at three different lengths for the V3 region. The three sets are presented with solid lines, and the OTU cluster compartments in dashed lines in matching colors. (**D**) Set resilience to clustering following an increasing sequence identity threshold. The x-axis shows an increasing identity threshold for clustering exercises. The y axis shows the percentage of sequences that remain as unique centroids after each clustering iteration. The inner panel shows the 100–96% identity thresholds in more detail.

Despite the methodological differences, 927 of the cluster-sequences (12.96%) were identical among all three sets (Figure 2A). Of this three-way overlap, their corresponding clusters accounted for 87.71% of all reads. Regarding two-way overlaps, there was also a relatively sizeable cluster-sequence overlap between both OTU sets (1,814 sequences, 12.96%), although their corresponding clusters accounted for only 2.63% of all reads in all three sets. However, 234 sequences were exclusive to the 02_99_OTU, and 03_ASV sets overlap but accounted for more reads (3.74%). Only a relatively small overlap occurred between the 01_OTU_97 set and the 03_ASV (0.12% of cluster-sequences and 0.36% of all reads). Most of the 11,005 set-specific cluster-sequences in this study were found in set 02_OTU_99 (8,566 cluster-sequences, 61.20%), but these accounted for a relatively small proportion of all reads (2.50%). Despite having the minor collection of centroid sequences (229), set 03_ASV summed more reads than the unique sequences in set 01_OTU_97 (2.31% and 0.75%, respectively).

To evaluate how each set of sequences compared to one another, is essential to notice that clusters in the OTUs sets may have two possible origins under QIIME’s recommended open-reference pipeline [40]. Either they might be derived from matching previously-clustered references, or produced by clustering amplicon reads at a fixed sequence identity cutoffs (*de novo*-based clusters). As Figure 2B shows, unique centroid-sequences in both OTU sets were from *de novo* clusters, most notably in the 02_OTU_99 set. However, in terms of total sequences, most reads were grouped into reference-based clusters.

Sequence variation within each set was evaluated as a proxy for the clustering resolution by determining how different each pair of sequences were to one another (Figure 2C). Single nucleotide changes included indels and mismatches, and the minimum sequence identity was set to 75%, a lower limit close to related phyla [14]. In total, 32.93% of all 03_ASV cluster sequences presented a single nucleotide difference, compared to 17.94% in 01_OTU_97 and 23.02% in 02_OTU_99, which were confirmed to be derived from reference-based clustering. In contrast, most *de novo* clusters did not match any other sequence until 4, and 2 nucleotide changes accumulated, respectively, which matches the limit of predicted 97% and 99% sequence identity for short and medium-length V3 16S rDNA sequences, as shown in the plot below. Whereas over 70% of all sequences in both OTU sets were less than 5 nucleotides apart, the 03_ASV centroids produced much fewer variants but were better outlined (the slope of the line in Figure 2C was not as steep and remained below the others as changes increased).

Since the identity percentage was different due to the varying length of each amplicon, a complementary analysis was carried out to determine how each set endured an increasing identity threshold for each pairwise permutations of its sequences. As seen in Figure 2D, the most similar sequences in different clusters had an identity of 99.2%, most of them observed in the 03_ASV set and to a lesser extent in both reference-based OTU sets. As expected, *de novo* 02_OTU_99 clusters had cluster centroids that shared up to 98.8% similarity, whereas the sequences in set 01_OTU_97 showed up to 97% similarity. Set 03_ASV set was the most resilient to clustering as it had the smallest number of sequences and a low proportion of homologous sequences in separate clusters.

### 3.3. Filters to Retain OTUs and ASVs, Accounting for >0.1% of the Total Abundance Per Sample

**Figure 3:**
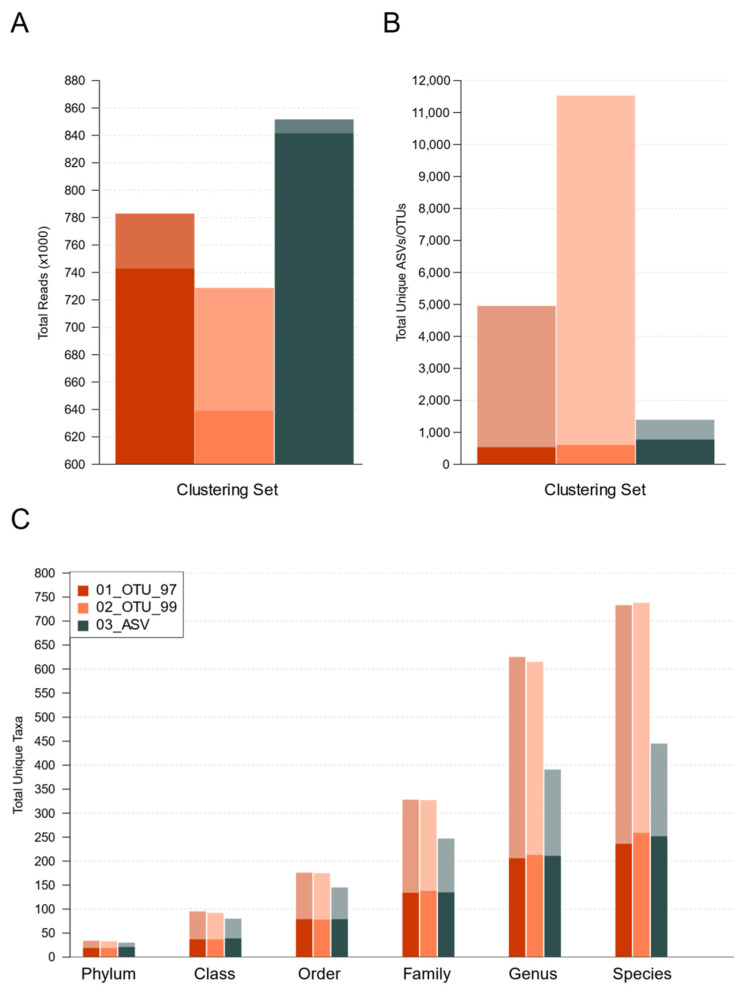
Impact of abundance filters on different performance parameters for each methodological set. Light-colored bars show the original raw totals, just after the removal of singletons and chimeras. Dark-colored bars show items remaining after abundance filters. (**A**) Total reads per set. (**B**) Total unique clusters (OTUs/ASVs) per set. (**C**) Total unique taxa per level and set.

In order to reduce artifactual variation among the samples and low-frequency clusters, we filtered all tables to retain the OTUs and ASVs, accounting for >0.1% of the total abundance per sample. This filter only impacted the OTU sets more strongly; however, it only decreased their number of total reads, with a loss of 5.16% and 12.32% of reads in sets 01_OTU_97 and 02_OTU_99, respectively, whereas only 1.21% of reads is ASV set (Figure 3A, dark bars; Supplementary Table S3.2). Precisely, both OTU sets were split into a larger number of total clusters than their ASV counterpart (Figure 3B, light-colored bars). However, the abundance filter effectively reduced variation in terms of unique clusters among all sets, which was leveled off at <800 different clusters per set (Figure 3B, dark-colored bars). OTU sets lost a much higher percentage of their clusters to filters, 89.27% in 01_OTU_97, and 94.78% in 02_OTU_99, since low-frequency or rare clusters were more abundant. In contrast, set 03_ASVs lost 44.99% clusters to filters (Supplementary Table S3.2), resulting in a higher number of unique ASV sets passing the filters (774) than unique OTUs (01_OTU_97: 533 and 02_OTU_99: 603).

The reduction of the unique OTUs and ASVs was also observed in the resulting taxonomy, which was noticeably leveled off at all levels (Figure 3C, dark bars). Interestingly, after the abundance filter, 58.33% phyla, 39.39% classes, 44.86% orders, 42.20% families, 36.17% genera, and 36.04% species of the total taxa remained independently of OTUs or ASVs method (Figure 3C, light-colored bars, and Supplementary Table S3.2). Thus, the total number of unique taxa and assigned taxonomies were similar between OTUs and ASVs after filter abundance application. All results hereafter refer to the resulting filtered sets.

### 3.4. Evaluating Taxonomy-Related Differences

**Figure 4:**
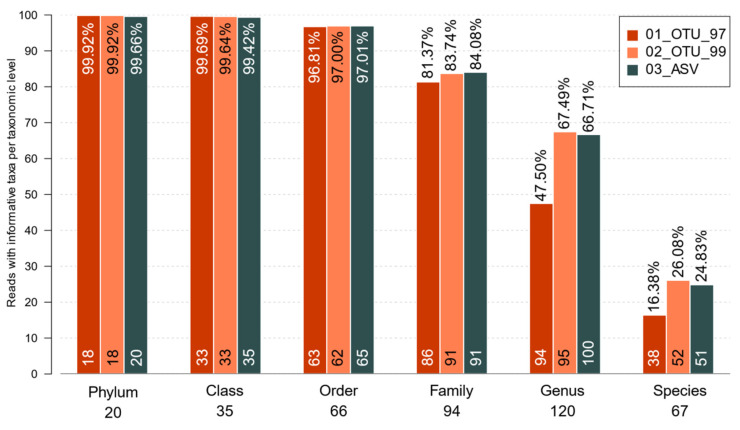
Percentage of reads bearing taxonomically-informative tags for the sets. Only taxa with a valid unambiguous terminal taxonomic node are included in each level. The read percentages are displayed at the top of each bar. Numbers below the taxonomy labels show the total informative tags per level, while numbers on the bottom of each bar show how many are found in each set.

Regarding the maximum taxonomic resolution, all sets produced a similar number of total taxa with informative (non-ambiguous) taxonomical names at each taxonomic level, showing limited success at the species level (Figure 4). All three sets assigned over 80% of all reads with a family-level resolution. It is important to note that the other 20% did have a valid taxonomy, but the centroid sequences of those clusters were similar to multiple phylogenetically-distant sequences. That resulted in a truncated taxonomy at a lower taxonomic level, where the last common ancestor (LCA) of the resulting hits would be found. A few of these, however, were truncated due to direct assignation to incomplete taxonomies derived from the 99% reference database OTUs that was used for an assignation. At genus-level resolution, the 47.50%, 67.49%, and 66.71% of sequences in the 97%, 99% identity clusters, and ASVs were assigned, respectively (Figure 4). Likewise, 26.08% and 24.83% of the reads were assigned at the species level for 0TUs_99 and ASVs, respectively, while only 16.38% of the reads were assigned for OTUS_97 (Figure 4). To delve deeper into the possible causes of whether no species could be assigned for most sets, we traced back the sequences previous to filtering to assay their matching taxonomy. It turned out that out of the total sequences in the 01_OTU_97 set, 60.85% of all missing species were produced by a lower LCA of the clusters, whereas 39.15% were due to references having no species label. Similarly, 63.77% of 01_OTU_99 sequences had been truncated due to the LCA and 36.23% due to missing labels. Contrastingly, only 51.57% of the sequences with no species in the 03_ASV set were due to LCA, whereas 48.43% were due to missing labels.

**Figure 5:**
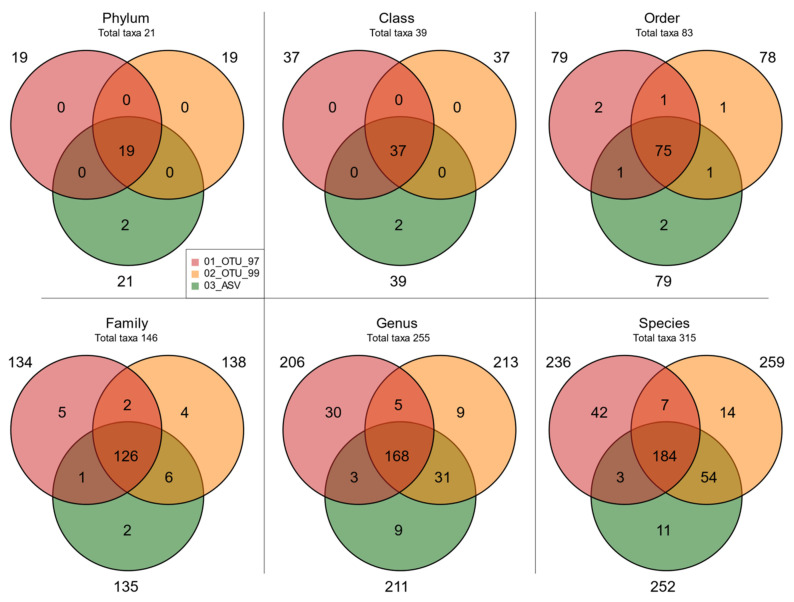
Taxa overlap in different sets. Venn diagrams of shared and unique taxa in the sets at different taxonomic levels. The total taxa per level are shown below the taxonomy label.

After that, we explored whether the obtained taxonomic profiles were equivalent. Venn diagrams in Figure 5 show a larger coincidence of matching taxa where 90.48% phyla, 94.87% classes, 90.36% orders, 86.30% families, 65.88% genera, and 58.41% species were detected by the three datasets. Most importantly, these taxa accounted for 99.99% reads in phyla and classes, 99.95% in orders, 99.77% in families, 97.40% in genera, and 94.95% in species (Supplementary Table S3.3). In contrast, taxa uniquely associated with a specific set represented <0.7% of reads regardless of the taxonomic level. It is also worth noting that the 03_ASV set had Archaea as their associated exclusive taxa, while set 01_OTU_97 showed the highest number of exclusive taxa; however, most corresponded to non-informative tags in higher taxonomic levels (unspecified genus and species). Interestingly, Venn diagrams of shared and unique taxa at different taxonomic levels prior to the abundance filter showed a high number of taxa associated with a specific set but with few sequences assigned (Supplementary Figure S2.2), suggesting the successful application of the abundance filter to homolog the taxonomy between clustering methods.

**Figure 6:**
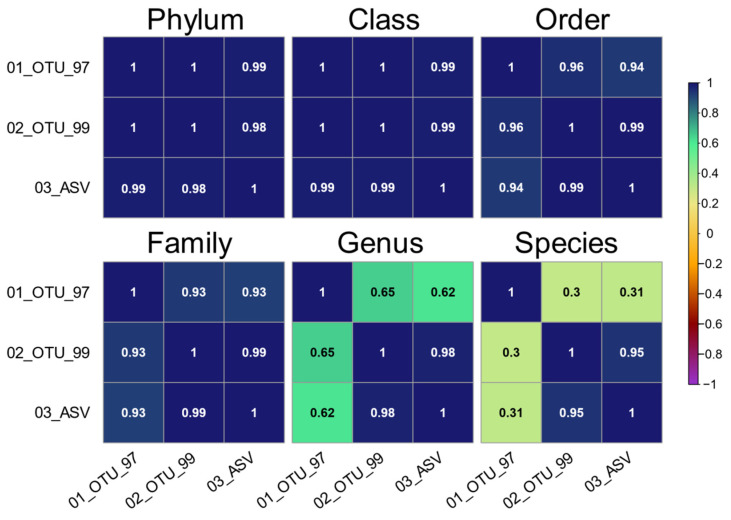
Spearman’s rank correlation (ρ) of the informative taxa distributions in all sets on multiple taxonomic levels. Only taxa with a valid unambiguous terminal taxonomic node are included. The upper or lower sections of symmetric matrices are presented per level to show all set permutations.

Next, we explored if the shared taxonomies were equivalent in terms of sequence abundance between the three sets. To this end, we analyzed the correlations between sets at all taxonomic levels, only considering taxa with informative tags (Figure 6). These were higher (ρ ≥ 0.93) from phylum through family levels between all three sets. The larger discrepancies were at the genus (ρ ≤ 0.65) and species levels (ρ ≤ 0.31) for the 01_OTU_97 set, showing higher correlations between the 02_OTU_99 and 03_ASV sets (ρ = 0.98 and ρ = 0.95, respectively).

We further explored whether taxonomic discrepancies between the 01_OTU_97 set, the most commonly used type of cluster used for studying shrimp microbiota, and the rest of the sets may arise from resolution differences. Taxonomic composition and abundance were similar up to the family level (Supplementary Figure S2.3), regardless of the clustering method as expected by the larger co-occurrence of taxa in such levels (Figure 5). Overall, Proteobacteria was the most abundant phylum, and Vibrionaceae, Methylobacteriaceae, Comamonadaceae, Caulobacteraceae, and Oxalobacteraceae the most abundant families (Supplementary Figure S2.3). Genus-level composition and relative abundance were congruent between the ASV and the 99% OTU-derived taxa but different from set 01_OTU_97.

The species-level showed a markedly different taxonomic distribution between the three sets, with the most pronounced differences in the 01_OTU_97 set (Figure 5 and Supplementary Figure S2.4). Approximately 25% of reads in this set were assigned to absent or low abundance species in the other sets (Supplementary Figure S2.4A), most importantly to undefined species from Vibrionaceae (17.42%). Other 56 such species represented 6.16% and included undetermined species from the order Burkholderiales and some bacilliary species only identified at order Gemellales. Contrastingly, 25.75% and 24.48% of all their reads for OTUs_OTU_99 and ASVs, respectively, were instead assigned to more specific species labels absent or in low abundance in the 01_OTU_97 set (Supplementary Figure S2.4B). These included undetermined species from genus *Vibrio* (10.72 ± 0.52% of reads in those sets, and 1.91% in set 01), and those from *Vibrio tapetis* (7.36 ± 0.28% in sets 02-05 compared to 0.83% in set 01). Other such 74 species collectively represented a cumulative abundance of 7.04 ± 0.16% in these sets, whereas, in set 01, these account for a much lower 0.21%. They included different species from Bacillales and Burkholderiales orders, and the Oxalobacteraceae family, *Pseudoburkholderia malthae*, *Massilia niastensis*, and an undetermined species of *Massilia*.

### 3.5. Collated Group Richness and Entropy Evaluated through α-Diversity

**Figure 7:**
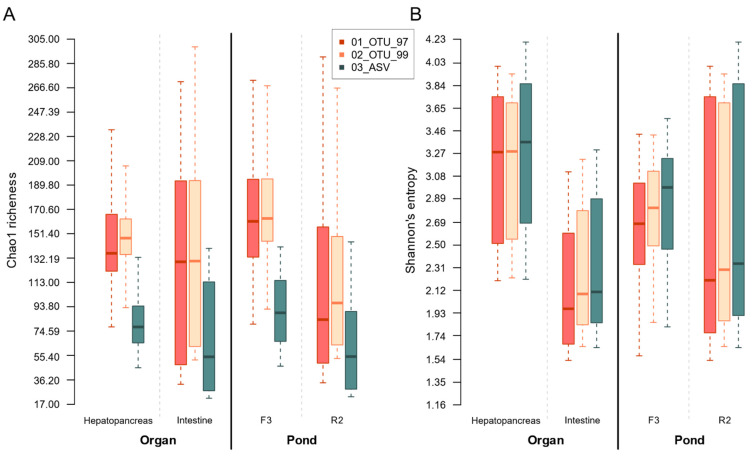
Group-collated within-sample diversity estimations for all sets. Each sample’s estimator was calculated from independent rarefactions drawn from each methodological set. Boxplots were drawn from the full array of observations per group. (**A**) Chao1 estimated richness adjusted for doubletons. (**B**) Shannon’s entropy.

We selected two organs, the intestine, and the hepatopancreas, to test a biological variable and two ponds, F3 and R2, to test an environmental variable in the effects on within-sample (α) and between-sample (β) diversity. Thus, for further analysis, we separated the samples according to their organ and pond source. We found that all three sets identified similar α-diversity trends for organ and pond, independently of the clustering method (Figure 7). Interestingly, both OTU sets showed a significantly (α = 0.05) greater expected richness (Chao1) than the 03_ASV set (Figure 7). On the contrary, the ASV set presented lower, albeit non-significant, diversity (Shannon’s entropy), suggesting that the OTU sets have a larger population of clusters with smaller frequencies that inflate the expected richness, whereas there are a slightly larger number of equally-distributed clusters in the ASV set. Comparison of the samples’ inner diversity (richness and entropy) at the different taxonomic levels revealed the same group differences observed in the diversity of OTUs and ASVs (Supplementary Figure S2.5).

Within each set comparison, the hepatopancreas samples had a greater, but non-significantly difference, expected richness (Chao1) and a greater and significant (α = 0.05) diversity (Shannon’s entropy) compared to the intestine samples (Figure 7), suggesting that three methods produce equivalent differences in within-sample diversity. We also evaluated an impact on the pond on α diversity microbiota where the expected richness difference between pond samples was significant in the OTU sets but not in the ASV set. In addition, the three sets reported significant differences between the entropy pond groups.

### 3.6. Group Abundance and Composition Differences Evaluated through β-Diversity

**Figure 8:**
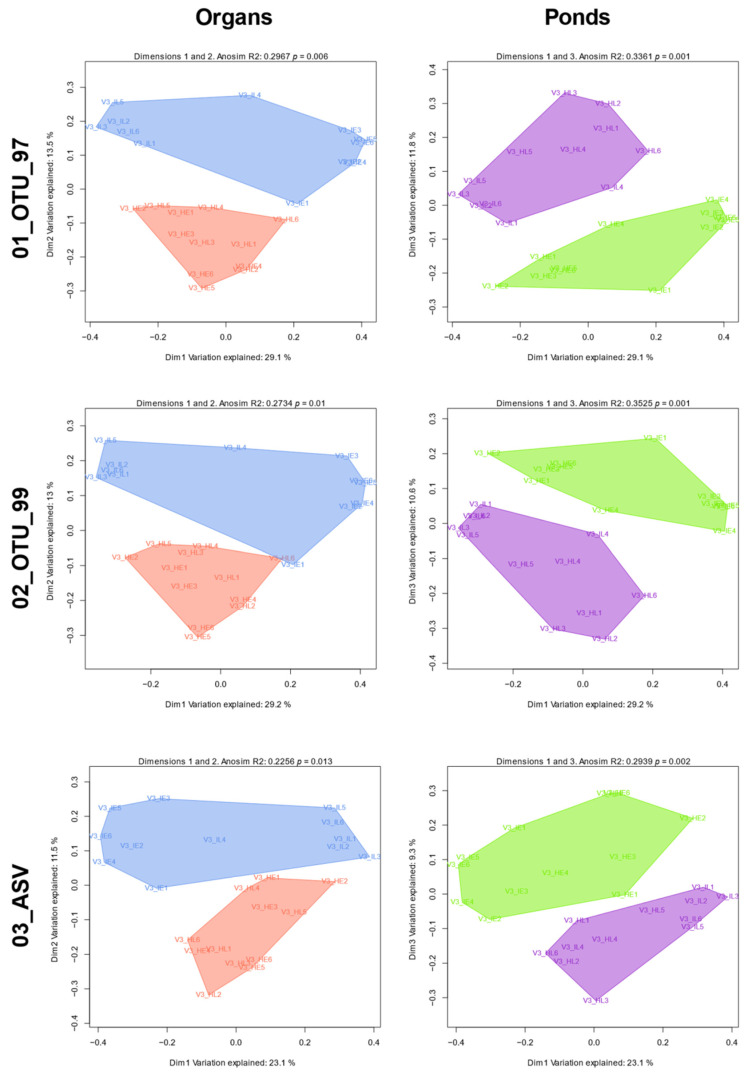
PCoA plots from phylogenetic-based composition set differences. Each 2D plot displays two of the first three linear clusters’ (OTU or ASV) combinations that best separate samples by each group configuration as derived from unweighted UniFrac (presence/absence) distance matrices per set. Polygons are shown per organ or pond but do not reflect actual clusters. Blue: Intestine; Coral: Hepatopancreas; Green: Pond F3; Violet: Pond R2.

All protocol sets were more homogeneous in between-sample diversity in terms of abundance than in composition and this was observed in all taxonomic levels. The unweighted UniFrac-derived PCoAs showed a sample separation between the intestine and hepatopancreas and between the F3 and R2 pond (Figure 8). ANOSIM R analysis showed that samples were homogenous in terms of composition between ponds (F3 and R2; R mean = 0.32 ± 0.03) and organs (I and H; 0.25 ± 0.02) for the three sets (Figure 8). When organ-pond groups were defined (IF3, IR2, HF3, and HR2), R statistics showed a higher and statistically significant (α = 0.05) group heterogeneity of both variables in shrimp microbiota for both the unweighted (R mean = 0.76 ± 0.63) and weighted (R mean = 0.60 ± 0.01) UniFrac distances in the three sets (Table 1). Lastly, we confirmed that diversity differences among groups were not restricted to cluster composition but reflected in each group’s taxa (Jaccard). Supplementary Figure S2.6 and Supplementary Table S3.4 showed that, except for phylum and class, all taxonomic levels showed statistically different (α = 0.05) samples among ponds and organs (as analyzed with ANOSIM). Although the sets had different power of resolution for diversity, they all produced the same patterns.

## 4. Discussion

The most significant differences in the overall bacterial composition in our study were those observed between the different organs, the intestine and the hepatopancreas. The homeostasis of aquatic species is heavily influenced by environmental conditions, such as temperature, salinity, pH, and nutrient availability impacting the diversity of microorganisms that share the same habitats [41]. The relevance of host-associated microbiota has been established by several studies focusing on the essential role that bacteria play in the intestinal tract. These bacteria play a role by modulating the immune response, establishing an ecological barrier by competing against pathogenic bacteria, and have critical roles in nutrient absorption and the regulation of metabolic processes [9,10,42].

Differences between ponds were also significant, which can be explained as the environmental microbial communities are particularly important in the establishment of the gastrointestinal microbiota, as the initial bacterial colonization is thought to have its origins in the surrounding water and sediment [4,43]. This has been supported by studies reporting similarities in the bacterial composition of sediment and that from the tract of aquatic species that present a burrowing behavior [41]. Even though rearing conditions are controlled in aquaculture crustacean production settings, the microbial composition has been found to vary between ponds in shrimp farms, but differences between ecological niches within the specimens are far more patent [4,12,44]. In this regard, studies in different shrimp species have reported a higher diversity in the hepatopancreas than in intestine samples, both of which are part of the gastrointestinal tract [12,45]. This may reflect an increased organ-specific selecting pressure, possibly due to its role in the immune response through the production of lectin, hemocyanin, ferritin, antibacterial proteins, proteolytic enzymes, and nitric oxide [46,47,48,49].

Currently, most studies exploring the 16S-profiles taxonomic composition of the gastrointestinal microbiota of aquatic species use identity-based OTU clusters to cope with the artifactual variability produced by high-throughput sequencing platforms. A practical and streamlined solution that groups 16S sequences is based on amplicon similarity between different taxa and varies in terms of sensitivity depending on the identity cutoff [14], as shown in Figure 1C. This was also seen in our study, where the 99% identity OTU set allowed for a more detailed taxonomic characterization than the 97% set of the less abundant amplicon variants, assumingly arising from less prevalent taxa [24]. 

The VSEARCH algorithm used in this study used the same type of centroid-based clusters used in older QIIME1’s uclust sets. The 99% threshold was selected since DADA2’s authors claimed their algorithm achieved a single-nucleotide resolution [15]. Since the V3 region is approximately 127–168 nt long, this would represent roughly a 99% identity variation. However, the higher sensitivity of this approach also introduced a larger number of OTUs that actually pointed to the same extant sequences and taxa (Figure 2 and Figure 3). Denoising methods have gained momentum in recent years as an unconstrained alternative (not sequence identity-dependent) that does not pose as much a tradeoff of sensitivity and accuracy since they aim at classifying each amplicon variant separately, considering the errors that may have been introduced during the sequencing [16,17,18,19,20,24]. Due to the growing popularization of denoising methods, we deem it necessary to study how future ASV-based profiles of aquatic species’ microbiota will compare to extant OTU-based studies. The DADA2 algorithm was selected as it has good technical support and receives regular updates from its creators. It is free (unlike UNOISE3) and has no sequence length limitations (as Deblur). As expected [15,18,19], the denoising approach used in the present study produced far fewer ASVs than OTUs but with higher abundances. (Figure 1C and Figure 3). Even though ASVs led to a limited number of species, working with less than 500 species is not necessarily detrimental but maybe, in fact, more accurate [19,20].

Rare cluster centroid variants in either the OTU or the ASV sets are where the three methods diverge. To study them, we analyzed the actual sequence variation and how their distribution varies between sets. The comparison showed that more than half of all unique centroid (representative) sequences were detected exclusively in the 02_OTU_99 set. Interestingly, these accounted for a mere 2.50% of all reads in the three sets (Figure 2A). Contrastingly, the much smaller overlap of unique centroid sequences existing between the three sets (6.62% of all centroid sequences) accounted for over 87% of all reads, which show that all three methods work well with the abundant amplicons.

Differences that may be attributed to the varying methodologies could be traced back to punctual changes (detected as indels or mismatches between all pairwise permutations) in the nucleotide sequence of amplicons sharing high sequence similarity (Figure 2C). Based on the results, set 03_ASV showed the highest proportion of high-resolution centroid sequences, with single-nucleotide level differences in almost twice as much as the percentage observed in set 01_OTU_97, all of which were found only in reference-based clusters. In contrast, set 02_OTU_99 showed the highest sequence-redundancy, as close to 70% of all sequences in that set differed in only two changes between one another, and more than 60% of them could be clustered at 98% sequence identity and 80% at 97% identity (Figure 2D).

Despite having a smaller number of total reads than the ASV set, reads in both OTU sets were grouped into a larger collection of clusters, each bearing a different unique centroid sequence (Figure 2B), more noticeable in the 02_OTU_99 set. This was expected, as rising the clustering identity assigned sequences into less populated clusters, resulting in an overestimation of the actual diversity. Even differences of just a couple bases may produce additional separate OTUs, compared with the 97% identity set. On the contrary, the ASV sets had fewer clusters (Figure 2B, light bars), as was expected due to denoising, which has been suggested to be a more accurate measure of the actual diversity [18].

In terms of maximum resolution, both the 99 OTUs and the ASVs had over 98% sequence identity in their more similar sequences. Interestingly, it turned out that using reference-based clusters in both OTU sets produced a few clusters bearing a similar resolution to that seen in the 03_ASV set (Figure 2C,D), while *de novo* clusters had the expected identity (97% or 98.8%) in the corresponding 97 and 99 sets. The upper limit of detection achieved in the 03_ASV set and the reference-based clusters appeared to be at 99.2% sequence similarity, representing a variation of approximately 1 nt in a 135 nt sequence. From these analyses, we concluded that set 03_ASV was the most resilient set, meaning that its clusters are well-outlined, show a similar resolution to that observed in the 02_OTU_99 but bearing a much lower cluster redundancy.

It is key to highlight that the main focus of the current study was not to evaluate the technical challenges of producing either identity clusters or denoising, nor how well they capture the known diversity. This is because most contemporary studies on aquatic ambiances still use OTUs, and specialized revisions have already demonstrated the major advantages and limitations of denoising methods [15,16,18,19,20]. Instead, we intended to determine how both types of profiles compare to one another if past and future studies may be compatible and may produce comparable shrimp taxonomy and diversity profiles. Most studies comparing the resulting taxonomies between OTUs and ASVs have focused on niches such as soil, mouse feces, human milk, and intestinal samples [19,21,22,23], but aquatic species had remained largely unexplored.

Regarding taxonomy, as we concluded in a previous study, the Greengenes database has some limitations for 16S profiling [12], mainly because it is no longer being maintained, but it was selected for this study for comparison to legacy sets as it is still the most commonly used reference in studies focusing on aquatic ambiances. Some of the issues in the database (common in the vanilla version of QIIME1) have been addressed as the cluster references have been recalculated in latter versions of QIIME2 with a newer Bayesian classifier algorithm [23], and we also fixed known issues with *Pseudoalteromonas* and *Vibrio* assignations. We found that filtering low-frequency clusters produced highly comparable microbiota sets in terms of taxonomy, α and β diversities (Supplementary Figure S2.5 and S2.6). This shows that existing OTU sets may be compared under even conditions to upcoming ASV sets by providing adequate sequence filters because variation in OTU sets is mainly comprised by large collections of small clusters whose centroid sequences vary in just a few nucleotides from those in larger clusters, which are successfully by all three approaches.

The frequency filters affected the 99% identity set more prominently, as a larger proportion of sequences were in low abundance OTUs (Figure 3B). This phenomenon derives from two main components that have been discussed in previous studies: first, the high sequence identity cutoff makes biological and artifactual variants indistinguishable, resulting in an overestimation of the total clusters [50,51,52]. Second, each 16S hypervariable region has a distinct taxonomic resolution [14], and the identity cutoff for higher levels (genus/species) is not evenly defined for all clades [12,53]. In our study, we have no means of determining the actual biological composition. However, our ASV set showed an increased resolution slightly higher than that of the 99% identity OTUs but with far fewer variants that differ in 1 or 2 bases as the ASV cluster centroids were more distant from one another(Figure 2C,D), as a result of the denoising process [15]. Even though we cannot assume that this is in fact due to overestimation in our case, it is clear that most of the additional sequences in the 02_OTU sets group into low-frequency clusters that are peripheric to the most abundant ones as would be expected for artifactual variants (Figure 1) and these would normally be discarded due to their low frequency. The taxonomic composition between the sets at all levels showed a high correlation between the 97% identity clusters and the other sets from phylum to family (ρ ≥ 0.93), which dropped at the genus and species levels (ρ ≤ 0.65, and ρ ≤ 0.31, respectively). Therefore, the comparison of taxonomic composition between the OTU and ASV sets is very feasible at most taxonomic levels (Figure 6), provided adequate frequency filters are included in the analyses. Notably, the taxa shared among the three datasets accounted for a high percentage of the total reads in all sets (99.76% in families, 97.40% in genera, and 94.95% in species).

The actual differences were seen in the abundance of ambiguous taxa, mainly found in the 97% identity OTU set. As we previously reported comparing hypervariable regions [12], most reads in the 97% OTU set from the V3 region can be unambiguously assigned to a specific family and nearly half of them to the genus level. However, higher-level clades often include several different species yielding non-informative assignations (Figure 4). Although the family-level resolution was similar for all sets (>80% reads), a significantly larger percentage of all reads had a genus-level resolution in the 99% identity and all ASV sets, along with a discrete increase in the species level, compared to the 97% OTU set. Thus, in the current study, in terms of taxonomic resolution, the ASV sets outperformed the 97% identity OTUs and matched the 99% set, albeit with cleaner cluster limits and more reads kept, as expected for denoising methods [15,16,17,19,20]. Regarding the reasons why clusters cannot be assigned at the species level, our exploration of the taxonomy associated with the centroid sequences revealed that ASVs had a lower proportion of non-assigned species that were due to the lack of consensus LCA than both OTU sets. In this set, barely half of the missing species could be explained by labels missing from the reference database.

Not all clusters that were derived from the different methodologies (OTUs/ASVs) produced the same sequences, as paired-end processing and sequence grouping are carried out differently between the methods, but the resulting taxonomic composition buffered these differences. In this regard, we observed similar numbers of taxa shared in all raw sets (Supplementary Figure S2.2, Supplementary Table S3.3) and similar abundance distributions between the 02_OTU_99 and the ASV set (Figure 6). Interestingly, the main difference between 01_OTU_97 and the 02_OTU_99 and ASV sets arises from taxonomic resolution (Figure 4 and Supplementary Figure S2.4), showing a larger proportion of phylogenetically distant clades in higher taxonomic levels in the former. For instance, whereas family Vibronaceae comprised >16% of the total reads in the 97% OTU set, these were instead assigned to genus *Vibrio* and species *V. tapetis* in the 99% OTU, similarly to what was reported for ASVs. *Vibrio* is a genus of Gram-negative bacteria from marine environments, which is pathogenic in culturable animals, such as *Vibrio alginolyticus* in fish [54], *V. tapetis* [55] in mollusks and *Vibrio parahaemolyticus*, and *Vibrio harveyi* in crustaceans such as prawn and shrimp [25,56,57,58]. In fact, the first study of shrimp microbiota using ASVs (carried out with UNOISE instead of DADA2) focused on specific *Vibrio* species and reported that the species’ diversity might be underestimated when using OTUs using direct sequence comparisons [25]. Similar observations were made with undetermined species from order Burkholderiales in set 01_OTU_97. The additional taxonomic resolution of 99 OTU clusters and ASVs allowed for the identification of *M. niastensis* and *Pseudoburkholderia malthae* instead. Gram-negative Burkholderiales were prevalent in the river and cultured shrimps [44,59], whereas genus *Massilia* comprise aerobic, motile bacteria that have been found in water, soil, and air and have been associated with nitrate reduction and chitin degradation [60,61]. Although its family, Oxalobacteraceae, has been reported to be prevalent in shrimp samples [44], there are few mentions of genus *Massilia* in related habitats [62] and none specifically of *M. niastensis* in these samples in the literature. A similar case occurs with *P. malthae*, a homotypic synonym of *Noviherbaspirillum malthae*, characterized as anaerobic, rod-shaped bacterium present in oil-contaminated sites [63] but has not been reported as part of shrimp microbiota. Together, these genera and species comprise 1–3% of the relative abundance in 99% OTUs and ASVs (Supplementary Figure S2.4) and show the difference in taxonomic resolution of ASVs, which will eventually require databases to include closer homologous references as the exact species found in shrimp may be missing.

All sets produced similar trends for richness and entropy for organs and ponds regardless of the ASVs or OTUs protocols. In general, richness was higher in hepatopancreas than in intestine samples, contrary to the Shannon entropy diversity (Figure 7). Taxa diversity comparison also showed similar tendencies (Supplementary Figure S2.5). This was quite positive since it suggests that previously published OTU surveys of shrimp microbiota may be compatible with future ASV assays in taxa and α-diversity analyses. The main difference with ASVs was that all the groups’ estimated values were lower, congruent to the smaller number of clusters and more homogeneous taxa grouping in the sets. Still, differences and proportions between organ and pond groups were a perfect reflection of those detected in the OTUs sets (Supplementary Figure S2.5).

Regarding β-diversity, ordination methods produced a full separation of samples, both by organ and pond, in all three sets using composition-based (unweighted) UniFrac distances (Figure 7). This further supports compatibility between the ASV and OTU methods, validating that both approaches can separate the microbiota from different organs and different ponds. Nevertheless, these groupings accounted for only a part of the explained variation. We also found that taxonomy can also effectively identify group differences at higher levels in both ASV and OTU sets (Supplementary Figure S2.6).

It is also clear that the host exerts robust filtering on establishing external microbes [64], and as stated before, both approaches show significant differences in the microbial communities between the shrimp gut and hepatopancreas [4]. Here, we observed a more substantial impact of the biological (organ) on microbiota structure than the environmental (pond) factor. Consistent with this pattern, it has been shown that sediment microbes are major sources for shrimp gut commensals in cultural pond ecosystems [65]. Also importantly, the microorganisms in shrimp aquaculture ponds could be associated with shrimp disease occurrence [66,67].

Regarding the limitations of the current study, we did not experimentally evaluate how representative our profiles were of the microbiome analyzed [68]. As microbial datasets are known to be compositional, experimental biases may be carried to downstream analyses [69]. This may have been addressed by the addition of a suitable mock community as a control to adjust for experimental biases. Yet, commercially available solutions are mainly focused on human-derived sites, and a separate design would be required in order to standardize a custom aquatic collection that included bacteria such as members from *Vibrio* species and from phyla Cyanobacteria and Verrucomicrobia, which are central to this niche, to take full advantage [4,44]. However, as the same set of sequences was used for creating all three profile sets, and since all samples were processed using the same protocol and sequenced together in a single run, the same amount of technical bias is expected to be homogenous in the whole set of sequences and hence in downstream analyses including clustering methods. Thus, we would not expect this to pose a critical limitation that may invalidate our results regarding comparability between methods.

Since the taxonomic composition and abundance in studies using OTUs and ASVs can be compared for shrimp samples under confined conditions used in this study, future prospect studies may include environmental samples from the rearing water and sediment.

## 5. Conclusions

We postulate that denoising techniques are indeed an alternative to identity clusters for 16S profiling, the current study describes to what extend both methodologies are comparable in terms of taxonomy, α, and β diversity profiles when exploring *L. vannamei*’s microbiota, as the advantages of ASVs have been amply explored elsewhere. An adequate preprocessing and filtering as the one proposed in this work will allow for a more even comparison between current shrimp microbiota studies that use OTUs and future studies using ASVs. The taxonomic resolution obtained by ASVs was very similar to the 99% identity OTUs, but having far less low prevalence clusters, showing a promising new alternative for studying shrimp microbiota. Most importantly, the OTU and ASV clusters can produce comparable α and β diversity profiles using the described frequency filters, allowing the detection of analogous organ and pond group differences as long as adequate filters are applied in order to remove the least populated clusters and taxa.

## Figures and Tables

**Table 1:** ANOSIM results for group comparisons with phylogenetic-based composition and abundance differences.

Metric	Set	Org R	Pond R	Org-Pond R	Org Pval	Pond Pval	Org-Pond Pval
Unweighted Unifrac	01_OTU_97	0.297	0.336	0.802	0.006	0.001	0.001
Unweighted Unifrac	02_OTU_99	0.273	0.352	0.805	0.01	0.001	0.001
Unweighted Unifrac	03_ASV	0.226	0.294	0.692	0.013	0.002	0.001
Weighted Unifrac	01_OTU_97	0.168	0.165	0.607	0.022	0.031	0.001
Weighted Unifrac	02_OTU_99	0.166	0.153	0.592	0.028	0.034	0.001
Weighted Unifrac	03_ASV	0.159	0.159	0.596	0.022	0.028	0.001

## Data Availability

Sequencing data sets are available in NCBI’s SRA under Accession Numbers: SRR11657998-SRR11658026.

## Supplementary Materials

The following are available online at https://www.mdpi.com/article/10.3390/genes12040564/s1, File S1: Supplementary Methods, File S2: Supplementary figures (Supplementary Figure S2.1: Dataset creation workflow, Supplementary Figure S2.2: Taxa overlap in different sets prior to frequency filters, Supplementary Figure S2.3: Relative abundance of all sets at the phylum, family and genus levels, Supplementary Figure S2.4: Relative abundance of species prevalent in the 97% identity cluster set and in the other sets, Supplementary Figure S2.5: Medians of within-sample estimators collated per group of samples across different taxonomic levels and sets, Supplementary Figure S2.6: Level of significance of differences between composition-based group distances), File S3: Supplementary tables (Supplementary Table S3.1: Preprocessing and clustering totals per sample and set, Supplementary Table S3.2: Total reads and unique features per set, Supplementary Table S3.3: Reads in clusters and taxa unique to each set). Supplementary Table S3.4: ANOSIM results for group comparisons with multiple metrics.
