# Supplementary material for: OTUs and ASVs Produce Comparable Taxonomic and Diversity from Shrimp Microbiota 16S Profiles Using Tailored Abundance Filters
Source: Genes (Basel). 2021 Apr 13;12(4):564. doi: 10.3390/genes12040564 (PMC8070570; doi:10.3390/genes12040564)
Supplement: Supplementary file 1 [file genes-12-00564-s001.zip › genes-1148811-supplementary/Supplementary File 1 - Additional Methods.pdf]

Additional Material and Methods

Bioinformatic procedures

Three different sequence sets were created to evaluate different bioinformatic processing protocols of the V3 amplicon reads (Figure S2.1).

Preprocessing

All fastq files with the resulting sequencing reads (hereafter "reads") were demultiplexed with CASAVA 1.8 and undetermined reads were discarded.

Demultiplexed Illumina adapter sequences and primers used for PCR amplification were removed from the three region sets using the corresponding sequences with Cutadapt v2.0 [1]. Primer 338F sequence (5'-ACTCCTACGGGAGGCAGCAG-3') was trimmed (e=0.2;o=14) from R1 (forward sequence of each pair), and primer 533R sequence (5'-TTACCGCGGCTGCTGGCAC-3') was trimmed (e=0.25;o=14) from R2 (reverse sequence of each pair).

Existing partial sequences of Illumina Nextera adapters were removed from the 3'-end of all sets using the sequences of non-biological constructs with the structure: 16S primer-nextera_adapter-index-sequencing_primer, allowing for a variable length. Construct 5'-CAGCAGCCGCGGTAACTGTCTCTTATATACATCTCCGAGCCCACGAGACN{8}ATCTCGTATGCCGTCTTCTGCTTG-3' was trimmed (e=25;o=21) from R1, and construct 5'-CCTCCCGTAGGAGTCTGTCTCTTATACACATCTGACGCTGCCGACGAN{8}GTGTAGATCTCGGTGGTCGCCGAATCATT-3' was trimmed (e=25;o=21) from R2. The correct trimming of the 16S primers was confirmed by a flanking sequence analysis with PRINSEQ v0.20.4 [2].

The sequences of sets 01_OTU_97 and 02_OTU_99 were quality-filtered using PRINSEQ that included a low-entropy sequences filter (entropy < 68%; including homopolymers and spurious repeats), trimming of low quality 3' (3 nt window, step=3, type=mean, q=20) and 5'-ends (5nt window, step=3, type=min, q=20) and removal of sequences with low average quality (q=22). Short-sequences (< 120 pb) were eliminated. The sequences from sets 01 and 02 were subsequently joined COPE v1.2.5 [3] with match ratio cutoff of 0.25, overlap range of 33-135 and insert of 195, and only joined sequences were considered for downstream analyses. Set 03_ASV was instead created from the Cutadapt-filtered set by filtering Paired-End sequences with DADA2 v1.12 suite in R programming language v3.6.0 [4,5]. Only sequences with max expected errors of 3 in R1 and 3.5 in R2 were kept. The set was comprised of previously joined reads and was treated as a single-end read set, filtering reads outside the 120-170 nt range or max expected error > 5.

OTU (identity) Clustering

The COPE-joined sets were used for the construction of Operational Taxonomic Unit (OTU) for sets 01_OTU_97 and 02_OTU_99, with the QIIME2 v2019.1 suite [6] as follows: Joined sequences were dereplicated with Vsearch v2.7.0 [7]. Open-reference clustering was then carried out at 97% identity for set 01 and at 99% identity with the corresponding clusters of the rRNA gene databases Greengenes 13_5 directed at the V3V4 regions [8] for reference-based clustering and Vsearch for *de novo* clustering. Singletons were removed and results were summarized in contingency tables. The resulting sets are hereafter referred to as 01_OTU_97 and 02_OTU_99.

ASV Clustering (denoising)

For the creation of the rest of 03_ASV, DADA-preprocessed reads were used for the construction of Amplicon Sequence Variants clusters (hereafter, ASVs) with DADA2 in R. Error models were created with all sequences over a maximum of ten iterations followed by sample-independent ASV creation. Paired-End reads were further joined with DADA2 according to their region overlap. Set 03_ASV, was created with min 50 nt overlap and ASVs with 130-159 nt were kept. Singletons were removed from all sets.

Chimera filtering

To homogenize the downstream analysis, all sets were subjected to the same set of downstream filters, first removing sample-based singletons (1s in the table, since ASVs are calculated independently by samples), then using Vsearch with scripts from the QIIME2 suite: Clusters were first subjected to detection of reference-based chimera filters using representative sequences from the Broad Institute gold database [9]. *De novo* chimeras were then predicted and the intersection of both the reference-based and *de novo* chimeras were removed.

The OTU sets, 01_OTU_97 and 02_OTU_99, were filtered so that all singletons in any sample were removed (to match inner DADA2 procedures carried out with the rest).

Taxonomic identification

Using 97% and 99% Greengenes clusters as reference, the V3V4 region fragments were extracted using primer sequences 341F and 805R to train the scikit-learn classifier v0.19.1 [10] for taxonomic identification. Raw reads were then subjected to taxonomic identification with the scikit-learn classifier with QIIME2 scripts. Only set 01_OTU_97 was identified with 97% identity clusters while the rest were identified with 99% identity clusters. QIIME2 scripts were used for collation of each taxonomic level per set and exporting as contingency tables. These tables are henceforth referred to as “Raw sets”.

Core-feature selection

Each OTU/ASV and taxonomic table was subjected to a filter to reduce the prevalence of empty observations (data sparsity) by discarding rare or low-frequency clusters/taxa. Only features (OTUs, ASVs or taxa) comprising at least 0.01% of the total abundance in any sample were retained (they were kept throughout the table if the conditions were met for any given sample). The filters were implemented with in-house R scripts and the sparsity reduced tables are henceforth referred to as “Filtered sets”. Feature thresholds were applied as a percentage of all sequences in a given sample (locally), instead of all sequences within the whole dataset (globally) in order to avoid filtering biases because it could not be assumed that the present populations were homogenous nor that they had comparable abundances [11].

Comparing the performance of OTU and ASV sets

Raw and core sets were analyzed in terms of total reads per set and total unique clusters and taxa in each taxonomic level using in-house R scripts. Also, each table was evaluated to determine the total number of informative taxa (not having an empty terminal node) in each taxonomic level as a way to compare the resolution level of each set. Additionally, R scripts were used to calculate Spearman’s rank correlations comparing the cumulative abundance in all three sets of each taxonomic level using the informative taxa of the core sets.

For the sets, the contents of each taxonomic level per whole set was collated with R scripts and compared in three-way Venn diagrams showing total present unique taxa shared between the sets. Differences with the 01_OTU_97, which is currently the most commonly used type of protocol in this field of shrimp microbiota, were assessed by comparing taxa in sets 02-03 showing ≤33% the abundance of that reported by the 01_OTU_97 set (taxa that were diminished in the other sets with respect to the 97% OTU set) and the taxa in each set that where the 01_OTU_97 set had ≤33% the abundance (those increased in other sets with respect to the 97% OTU set) which was calculated by using the 03_ASV as reference as it is methodologically closer to the OTU sets.

Within-sample, α diversity comparison

Shannon entropy, the total observed features (OUT/ASV/taxonomic level), and expected Chao1 richness were estimated with vegan (v2.5-6) in R [12] for each sample, set and OUT/ASV/taxonomic level. This was carried with Montecarlo repetitions consisting on the mean observations over 10,000 rarefactions of the raw and filtered tables at the depth of the smallest sample to cope with uneven sample depth (hereafter "standardized tables"). Observations per sample were statistically compared across all repetitions using medians. Samples were compared by Organ (hepatopancreas or intestine, H and I), Pond (F3 or R1), and Org-Pond (HF3, IF3, HR2, IR2). The resulting values and their statistical comparisons were collated into a matrix depicting how each sample and group differences per set and presented in heatmaps. Statistical significance was considered at an α = 0.05 throughout the study.

Between-sample, β diversity comparison

For each OTU/ASV/taxonomic level in the Filtered sets, standardized tables were created as described above. A Jaccard (absence/presence) and Bray-Curtis (abundance) dissimilarity matrices were constructed from the resulting standardized contingency tables using vegan and in-house R scripts. Each dissimilarity matrix was then subjected to a Principal Coordinate Analysis (PCoA) Ordination Method to evaluate group separation considering the first two linear combination of features. Anosim non-parametric test were used to compare between the groups (as defined in the previous section) using pairwise group tests for ad-hoc testing. All results were evaluated with the construction of heatmaps (R) bearing all sets and taxonomic levels.

Filtered tables were further used to construct UniFrac matrices bearing phylogenetic information. For this, the respective standardized tables were used to subset their corresponding representative sequences. These were in turn used for a rooted phylogenetic reconstruction with SEPP [13], using QIIME 2 scripts. For both OTU sets, a manual fix was required to prevent branches from having the same names as SEPP's reference (GG 99). The resulting tree was used for calculation of weighted and unweighted UniFrac distance matrices for each set in the Filtered groups. The resulting matrices were then used to carry the same analyses used for Jaccard and Bray-Curtis matrices as described above.

Data access

Sequencing sets were deposited in NCBI's SRA and are available via online with Accession Numbers: SRR11657998-SRR11658026.

1. Martin, M. Cutadapt removes adapter sequences from high-throughput sequencing reads. *EMBnet.journal* **2011**, *17*, 10, doi:10.14806/ej.17.1.200.

2. Schmieder, R.; Edwards, R. Quality control and preprocessing of metagenomic datasets. *Bioinformatics* **2011**, *27*, 863–864, doi:10.1093/bioinformatics/btr026.

3. Liu, B.; Yuan, J.; Yiu, S.M.; Li, Z.; Xie, Y.; Chen, Y.; Shi, Y.; Zhang, H.; Li, Y.; Lam, T.W.; et al. COPE: An accurate k-mer-based pair-end reads connection tool to facilitate genome assembly. *Bioinformatics* **2012**, *28*, 2870–2874, doi:10.1093/bioinformatics/bts563.

4. R core team R: A Language and Environment for Statistical Computing Available online: https://www.r-project.org/.

5. Callahan, B.J.; McMurdie, P.J.; Rosen, M.J.; Han, A.W.; Johnson, A.J.A.; Holmes, S.P. DADA2: High-resolution sample inference from Illumina amplicon data. *Nat. Methods* **2016**, *13*, 581–583, doi:10.1038/nmeth.3869.

6. Bolyen, E.; Rideout, J.R.; Dillon, M.R.; Bokulich, N.A.; Abnet, C.C.; Al-Ghalith, G.A.; Alexander, H.; Alm, E.J.; Arumugam, M.; Asnicar, F.; et al. Reproducible, interactive, scalable and extensible microbiome data science using QIIME 2. *Nat. Biotechnol.* **2019**, *37*, 852–857, doi:10.1038/s41587-019-0209-9.

7. Rognes, T.; Flouri, T.; Nichols, B.; Quince, C.; Mahé, F. VSEARCH: A versatile open source tool for metagenomics. *PeerJ* **2016**, *2016*, 1–22, doi:10.7717/peerj.2584.

8. DeSantis, T.Z.; Hugenholtz, P.; Larsen, N.; Rojas, M.; Brodie, E.L.; Keller, K.; Huber, T.; Dalevi, D.; Hu, P.; Andersen, G.L. Greengenes, a chimera-checked 16S rRNA gene database and workbench compatible with ARB. *Appl. Environ. Microbiol.* **2006**, *72*, 5069–5072, doi:10.1128/AEM.03006-05.

9. Kyrpides, N.C. Genomes Online Database (GOLD 1.0): A monitor of complete and ongoing genome projects world-wide. *Bioinformatics* **1999**, *15*, 773–774, doi:10.1093/bioinformatics/15.9.773.

10. Varoquaux, G.; Buitinck, L.; Louppe, G.; Grisel, O.; Pedregosa, F.; Mueller, A. Scikit-learn: Machine Learning Without Learning the Machinery. *GetMobile Mob. Comput. Commun.* 2015, *19*, 29–33.

11. Bokulich, N.A.; Subramanian, S.; Faith, J.J.; Gevers, D.; Gordon, J.I.; Knight, R.; Mills, D.A.; Caporaso, J.G. Quality-filtering vastly improves diversity estimates from Illumina amplicon sequencing. *Nat. Methods* **2013**, *10*, 57–59, doi:10.1038/nmeth.2276.

12. Jari Oksanen, F. Guillaume Blanchet, Michael Friendly, Roeland Kindt, Pierre Legendre, Dan McGlinn, Peter R. Minchin, R. B. O'Hara, Gavin L. Simpson, Peter Solymos, M. Henry H. Stevens, Eduard Szoecs, H.W. The Vegan Community Ecology Package 2019.

13. Janssen, S.; Mcdonald, D.; Gonzalez, A.; Navas-molina, J.A.; Jiang, L.; Xu, Z. Phylogenetic Placement of Exact Amplicon Sequences. *mSystems* **2018**, *3*, e00021-18, doi:10.1128/mSystems.00021-18.
