# Supplementary material for: OTUs and ASVs Produce Comparable Taxonomic and Diversity from Shrimp Microbiota 16S Profiles Using Tailored Abundance Filters
Source: Genes (Basel). 2021 Apr 13;12(4):564. doi: 10.3390/genes12040564 (PMC8070570; doi:10.3390/genes12040564)
Supplement: Supplementary file 1 [file genes-12-00564-s001.zip › genes-1148811-supplementary/Supplementary File 2 - Figures.pdf]

Supplementary figures

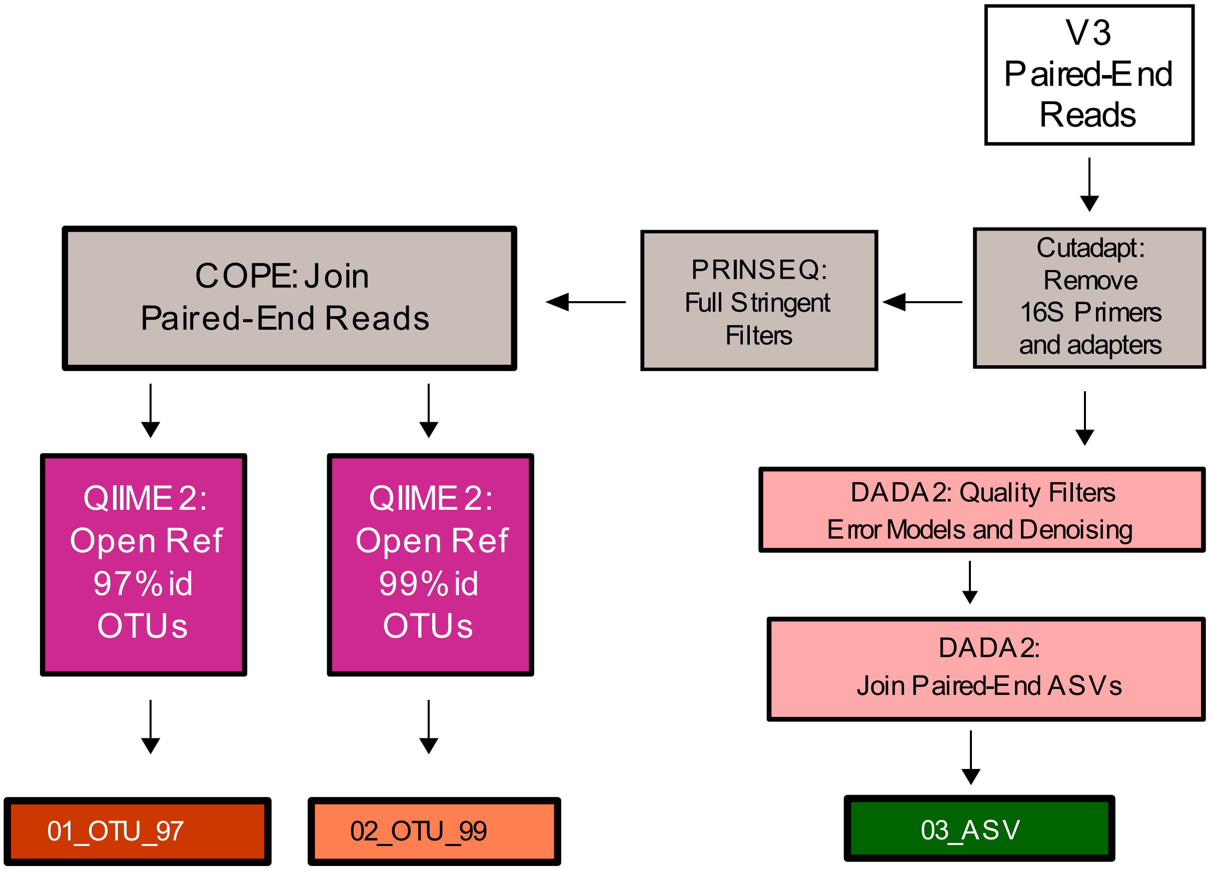

Figure S2.1. Dataset creation workflow. Preprocessing and clustering general methods employed for the creation of the three sets. The corresponding 16S primers were previously removed from all sequences. Sets 01 and 02 were carried out with an identity clustering scheme with QIIME 2 (97% and 99% identity, respectively). Set 01 was created using a standard OTU approach. Sets 03_ASV used a denoising scheme with DADA2.

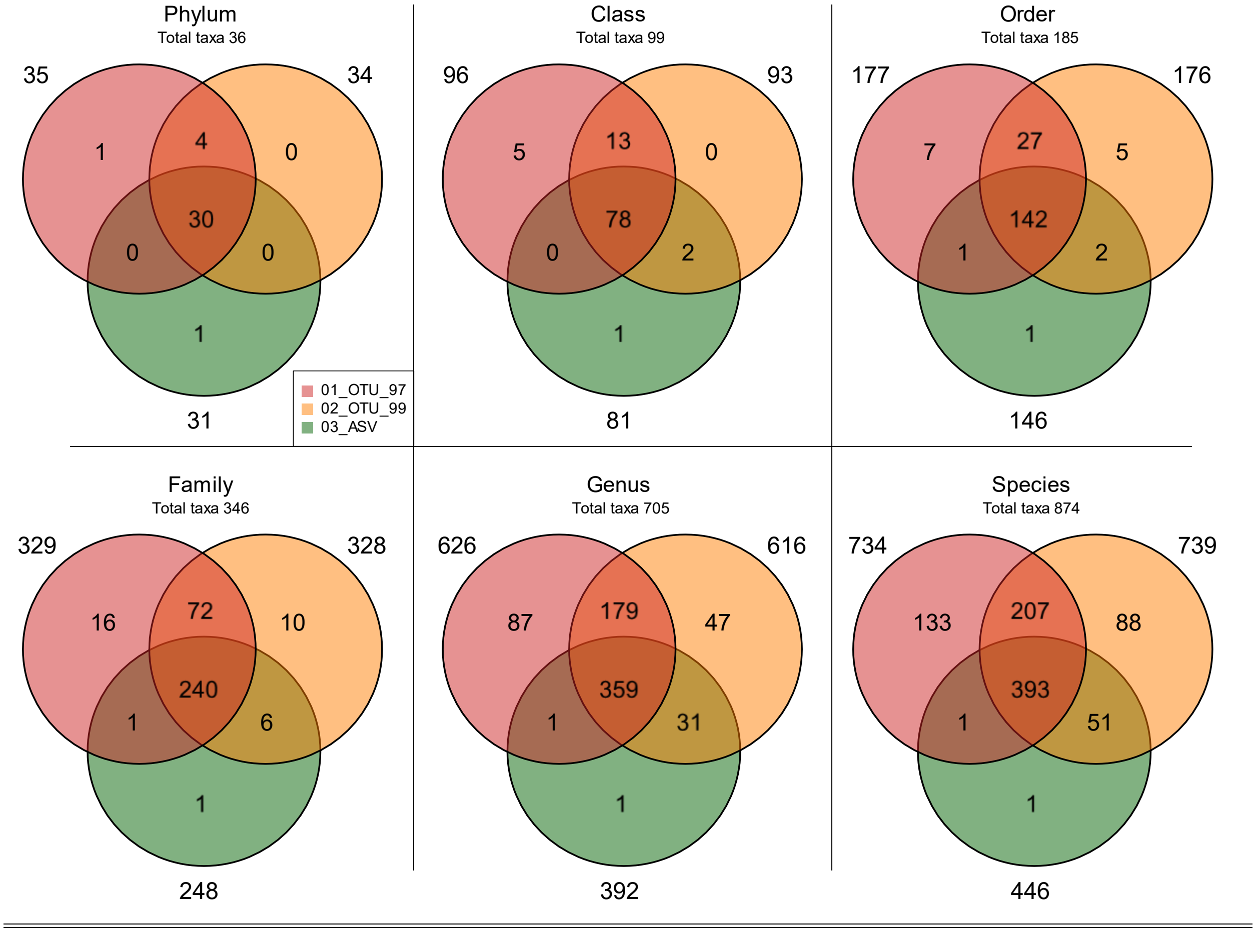

Figure S2.2. Taxa overlap in different sets prior to frequency filters. Venn diagrams of shared and unique taxa in the sets at different taxonomic levels prior to frequency filters.

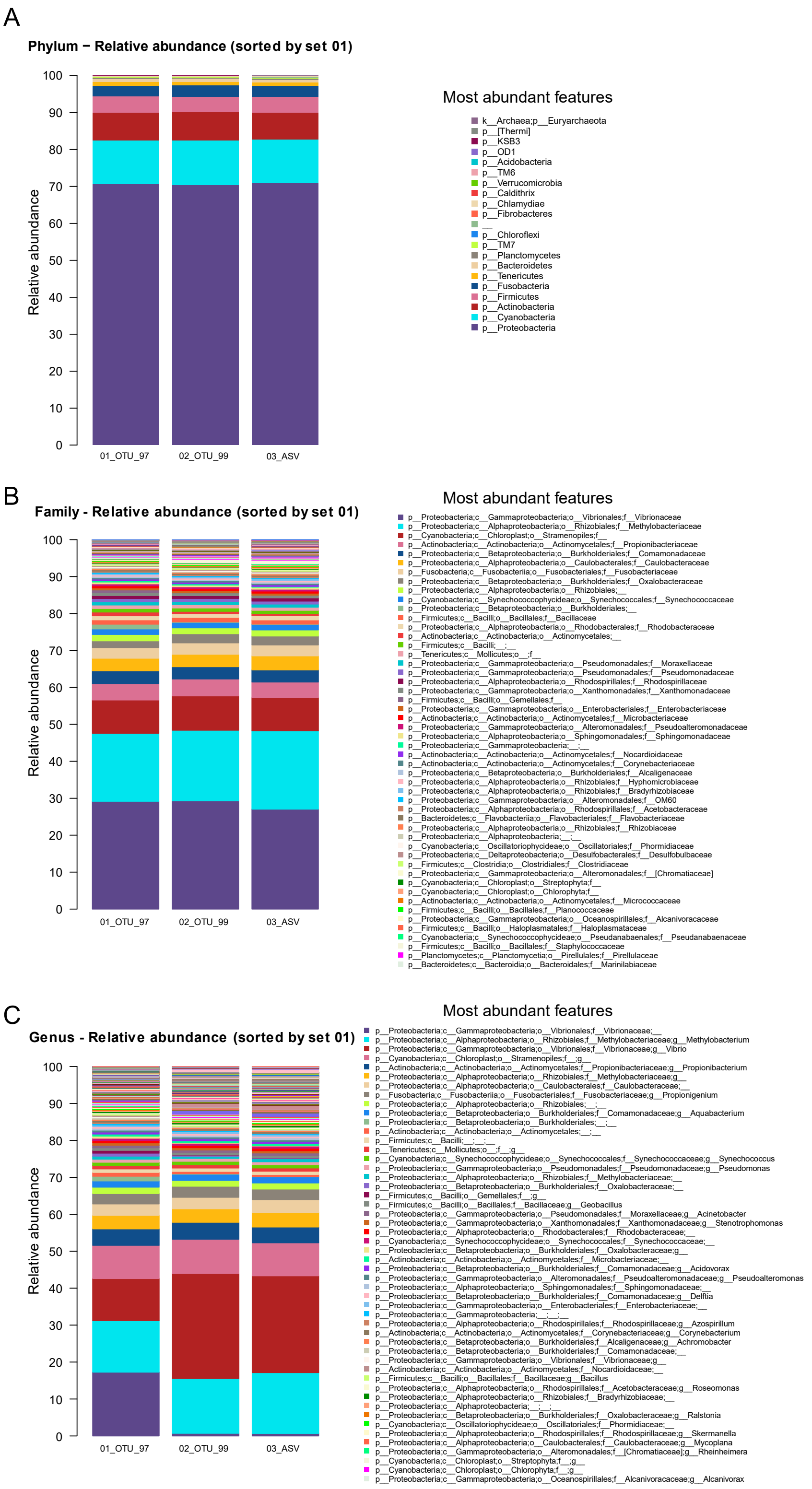

Figure S2.3. Relative abundance of all sets at the phylum, family and genus levels. Each column shows the abundance of each taxa sorted according to the distribution in set 01. A maximum of 50

most abundant features are shown on the right of its corresponding level. (A) Phylum. (B) Family (C) Genus.

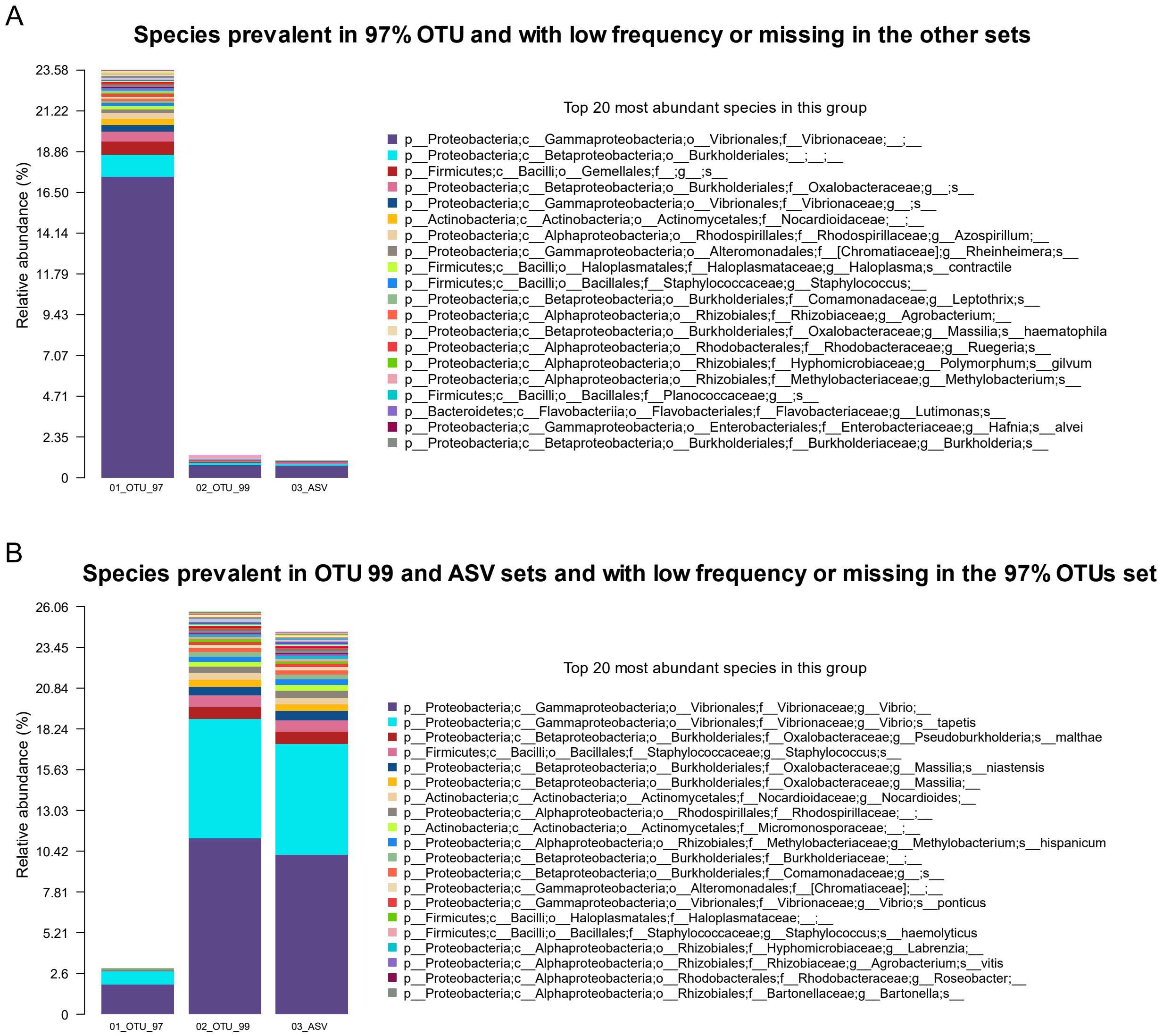

Figure S2.4. Relative abundance of species prevalent in the 97% identity cluster set and in the other sets. Shown species are more prevalent in either the 01_OTU_97 or the rest of the sets. These are mutually exclusive and respond to taxonomic resolution differences. Only the top 20 most abundant species are named. (A) Species that are more than three times as common in the

01_OTU_97 set with respect to the other sets. (B) Species that are more than three times as common in sets other than the 01_OTU_97 set.

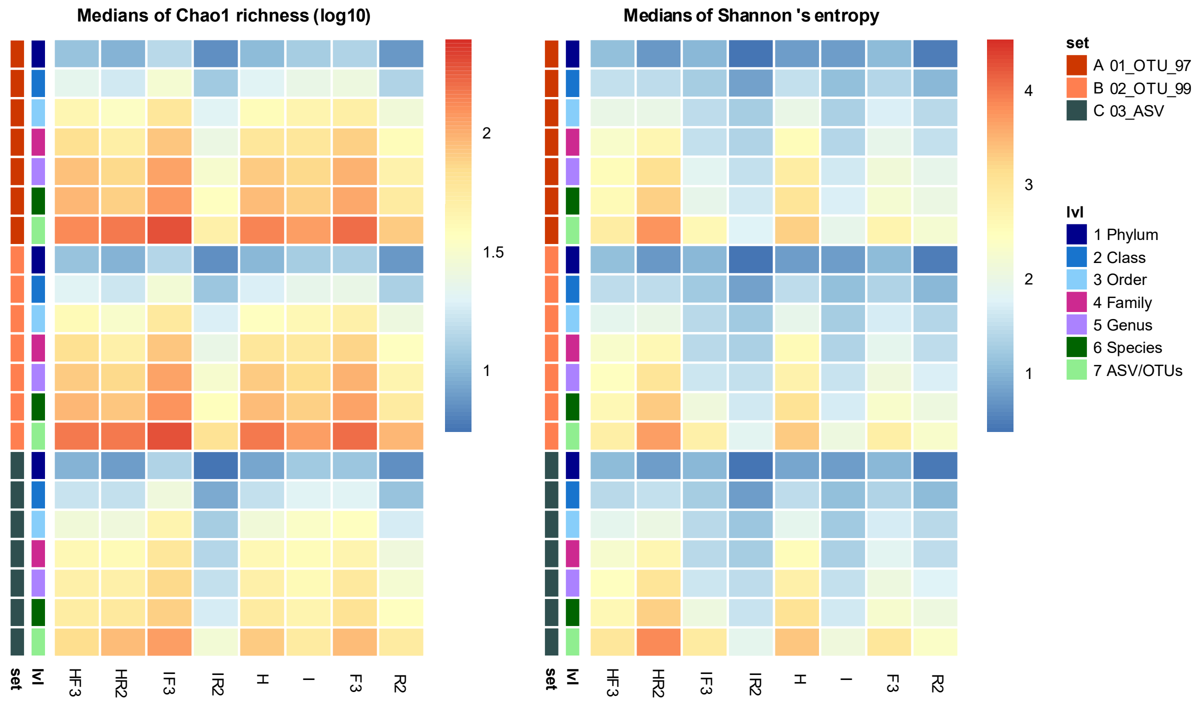

Figure S2.5. Medians of within-sample estimators collated per group of samples across different taxonomic levels and sets. Chao1 estimated richness and Shannon's entropy are shown for each group and combination. H: Hepatopancreas. I: Intestine. F3 and R2: different ponds. HF3 and HR2: Hepatopancreas from each pond. IF3 and IR2: Intestine samples from each pond. Chao1 is presented with $\log_{10}$ values for clearer comparisons.

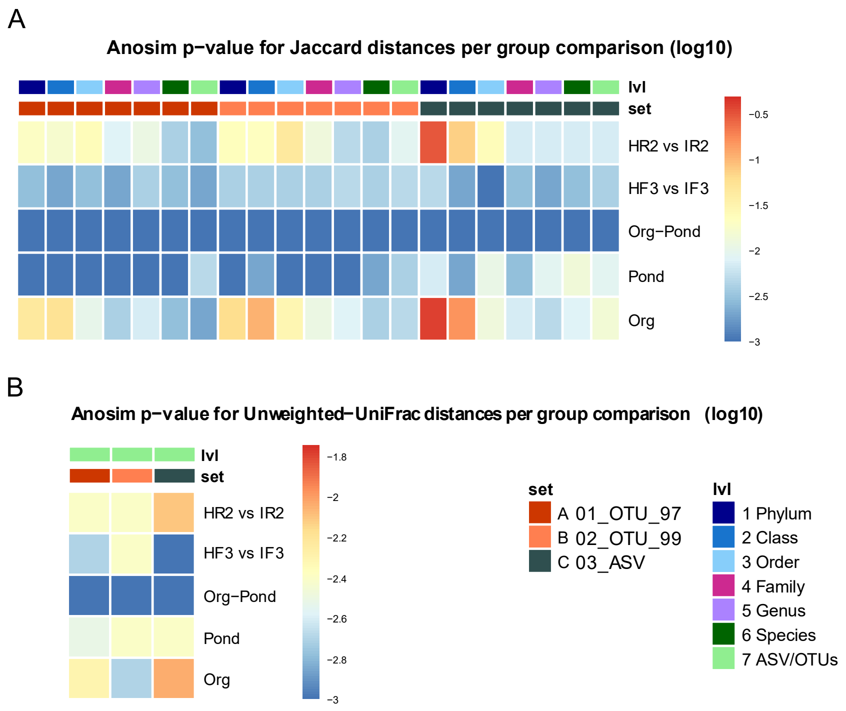

Figure S2.6. Level of significance of differences between composition-based group distances. (A) Anosim p-values for Jaccard distances (log_{10}) for the most relevant pairwise comparisons to determine the resolution power to detect differences between each group at all levels from phylum to species and clusters (ASVs/OTUs). (B) Anosim p-values for Unweighted UniFrac distances (log_{10}) for the most relevant pairwise comparisons to determine the resolution power to detect differences between each group, as calculated from the clusters (ASVs/OTUs). HR2 and IR2: Hepatopancreas and Intestine groups from pond R2. HF3 and IF3: Hepatopancreas and Intestine groups from pond F3.
