# Supplementary material for: OTUs and ASVs Produce Comparable Taxonomic and Diversity from Shrimp Microbiota 16S Profiles Using Tailored Abundance Filters
Source: Genes (Basel). 2021 Apr 13;12(4):564. doi: 10.3390/genes12040564 (PMC8070570; doi:10.3390/genes12040564)
Supplement: Supplementary file 1 [file genes-12-00564-s001.zip › genes-1148811-supplementary/Supplementary File 3 - Tables.pdf]

Table S3.1. Preprocessing and clustering totals per sample and set.

Sample	*Raw*	*Cutadapt Filters*	*Prinseq Filters*	*Cope Join*	*01_OTU_97*	*02_OTU_99*	*03_ASV*	*Ponds*
V3_HE1	61875	52509	51661	45716	45277	42294	48855	L=R2
V3_HE2	47716	39328	38527	31154	30844	28977	36448	E=F3
V3_HE3	46917	38897	38152	33779	32915	29955	34500	
V3_HE4	59493	51563	50593	42631	41792	38038	46864	
V3_HE5	35357	29369	28873	24390	23924	21689	26860	
V3_HE6	41102	37100	36416	29604	29327	27668	35084	
V3_HL1	43970	39962	39186	33787	32985	29629	36829	
V3_HL2	41333	35652	35090	30786	30189	26874	31984	
V3_HL3	50202	42142	41354	36973	36388	32566	37596	
V3_HL4	51624	43888	42926	38509	37949	34495	40172	
V3_HL5	57080	50540	49681	45251	44924	41511	47592	
V3_HL6	63120	55570	54615	48962	47885	42645	49398	
V3_IE1	47264	42645	41900	38350	37579	35161	38757	
V3_IE2	52704	50064	49302	46845	45973	42397	44518	
V3_IE3	43865	41989	41242	38142	37704	36059	39028	
V3_IE4	49797	39396	38666	35619	34988	32598	36084	
V3_IE5	45956	44159	43395	40140	39150	36557	39564	
V3_IE6	42767	40319	39517	35696	34700	32363	36178	
V3_IL1	39943	28142	27663	24322	24236	23047	27205	
V3_IL2	32353	24963	24321	20462	20337	19253	23447	
V3_IL3	34013	26609	26068	22176	22046	20703	25314	
V3_IL4	46732	43564	42881	41214	40966	39203	41638	
V3_IL5	29675	24119	23710	19815	19726	18765	23143	
V3_IL6	37712	34926	34336	29177	29044	27766	33207	
Mean	45940.4	39892.3	39169.8	34729.2	34202	31675.5	36677.7	
SD	8766.43	8592.2	8464.32	8282.39	8088.06	7335.93	7543.79	
Total	1102570	957415	940075	833500	820848	760213	880265	

Table S3.2. Total reads and unique features per set.

Class	*Order*	*Family*	*Genus*	*Species*	*OTUs/ASVs*	*Total Reads*
96	177	329	626	734	4968	783114
93	176	328	616	739	11541	728793
81	146	248	392	446	1407	854845
99	185	346	705	874		
Class	Order	Family	Genus	Species	OTUs/ASVs	Total Reads
37	79	134	206	236	533	742714
37	78	138	213	259	603	638996
39	79	135	211	252	774	844525
39	83	146	255	315		

Table S3.3. Reads in clusters and taxa unique to each set.

	Level	*Total Reads*	*Reads in all taxa*	*Reads only 01_OTU_97*	*Reads only 02_OTU_99*	*Reads only 03_ASV*	*Reads only OTUs*	*Reads only ASVs*
Raw	Phylum	2366685	2366608	2	0	56	2	56
	Class	2366568	2366258	60	0	56	60	56
	Order	2366344	2364883	65	35	56	100	56
	Family	2365991	2360320	112	46	56	158	56
	Genus	2365342	2323221	5092	406	56	5498	56
	Species	2365070	2297552	10851	877	56	11728	56
	Raw	Total Taxa	Taxa all sets	Taxa only 01_OTU_97	Taxa_only_02_OTU_99	Taxa only 03_ASV	Taxa Only OTUs	Taxa only ASVs
	Phylum	36	30	1	0	1	1	1
	Class	99	78	5	0	1	5	1
	Order	185	142	7	5	1	12	1
	Family	346	240	16	10	1	26	1
	Genus	705	359	87	47	1	134	1
	Species	874	393	133	88	1	221	1
	Level	Total Reads	Reads in all taxa	Reads only 01_OTU_97	Reads only 02_OTU_99	Reads only 03_ASV	Reads only OTUs	Reads only ASVs
Filtered	Phylum	2366355	2366144	0	0	211	0	211
	Class	2364270	2364059	0	0	211	0	211
	Order	2361938	2360664	299	52	211	351	211
	Family	2358518	2353069	901	271	211	1172	211
	Genus	2350804	2289694	10361	624	763	10985	763
	Species	2348976	2230270	14300	1134	871	15434	871
	Filtered	Total Taxa	Taxa all sets	Taxa only 01_OTU_97	Taxa_only_02_OTU_99	Taxa only 03_ASV	Taxa Only OTUs	Taxa only ASVs
	Phylum	21	19	0	0	2	0	2
	Class	39	37	0	0	2	0	2
	Order	83	75	2	1	2	3	2
	Family	146	126	5	4	2	9	2
	Genus	255	168	30	9	9	39	9
	Species	315	184	42	14	11	56	11

Table S3.4. ANOSIM results for group comparisons with multiple metrics.

Metric	Set	Lvl	Org R	Pond R	Org-Pond R	HF3 vs IF3 R	HF3 vs HR2 R	HF3 vs IR2 R	IF3 vs HR2 R	IF3 vs IR2 R	HR2 vs IR2 R	Org pval	Pond pval	Org-Pond pval	HF3 vs IF3 pval	HF3 vs HR2 pval	HF3 vs IR2 pval	IF3 vs HR2 pval	IF3 vs IR2 pval	HR2 vs IR2 pval
Unweighted Unifrac	01_OTU_97	OTU/ASV	0.297	0.336	0.802	0.933	0.782	0.687	0.817	0.893	0.767	0.006	0.001	0.001	0.004	0.004	0.005	0.003	0.007	0.005
Unweighted Unifrac	02_OTU_99	OTU/ASV	0.273	0.352	0.805	0.904	0.715	0.698	0.833	0.92	0.746	0.01	0.001	0.001	0.002	0.005	0.002	0.002	0.001	0.004
Unweighted Unifrac	03_ASV	OTU/ASV	0.226	0.294	0.692	0.867	0.452	0.585	0.693	0.887	0.604	0.013	0.002	0.001	0.002	0.01	0.006	0.003	0.003	0.005
Weighted Unifrac	01_OTU_97	OTU/ASV	0.168	0.165	0.607	0.896	0.617	0.317	0.606	0.824	0.448	0.022	0.031	0.001	0.002	0.002	0.012	0.003	0.003	0.016
Weighted Unifrac	02_OTU_99	OTU/ASV	0.166	0.153	0.592	0.88	0.583	0.296	0.582	0.802	0.456	0.028	0.034	0.001	0.003	0.01	0.01	0.003	0.004	0.016
Weighted Unifrac	03_ASV	OTU/ASV	0.159	0.159	0.596	0.898	0.624	0.259	0.615	0.806	0.441	0.022	0.028	0.001	0.001	0.003	0.017	0.003	0.003	0.019
Jaccard	01_OTU_97	Phylum	0.109	0.425	0.536	0.808	0.352	0.613	0.544	0.807	0.302	0.047	0.001	0.001	0.003	0.003	0.008	0.004	0.002	0.02
Jaccard	01_OTU_97	Class	0.101	0.401	0.51	0.826	0.228	0.744	0.441	0.875	0.465	0.0569	0.001	0.001	0.002	0.005	0.006	0.003	0.007	0.017
Jaccard	01_OTU_97	Order	0.176	0.338	0.515	0.902	0.36	0.543	0.575	0.733	0.411	0.01	0.001	0.001	0.003	0.003	0.003	0.006	0.004	0.025
Jaccard	01_OTU_97	Family	0.23	0.399	0.659	0.848	0.594	0.641	0.741	0.776	0.618	0.004	0.001	0.001	0.002	0.004	0.006	0.004	0.003	0.008
Jaccard	01_OTU_97	Genus	0.237	0.39	0.697	0.823	0.727	0.656	0.759	0.744	0.655	0.007	0.001	0.001	0.004	0.001	0.004	0.007	0.002	0.012
Jaccard	01_OTU_97	Species	0.258	0.383	0.722	0.856	0.761	0.682	0.777	0.785	0.677	0.003	0.001	0.001	0.003	0.006	0.002	0.003	0.004	0.004
Jaccard	01_OTU_97	OTU/ASV	0.296	0.298	0.803	0.95	0.861	0.644	0.857	0.885	0.744	0.002	0.005	0.001	0.002	0.002	0.002	0.001	0.004	0.003
Jaccard	02_OTU_99	Phylum	0.093	0.418	0.514	0.75	0.369	0.606	0.52	0.765	0.302	0.0629	0.001	0.001	0.004	0.003	0.007	0.001	0.005	0.023
Jaccard	02_OTU_99	Class	0.059	0.351	0.463	0.756	0.219	0.695	0.37	0.861	0.347	0.1089	0.002	0.001	0.004	0.015	0.003	0.004	0.004	0.022
Jaccard	02_OTU_99	Order	0.144	0.369	0.5	0.907	0.295	0.521	0.55	0.708	0.295	0.03	0.001	0.001	0.004	0.009	0.006	0.002	0.006	0.047
Jaccard	02_OTU_99	Family	0.233	0.404	0.644	0.868	0.552	0.572	0.777	0.799	0.543	0.012	0.001	0.001	0.004	0.003	0.004	0.004	0.001	0.013
Jaccard	02_OTU_99	Genus	0.234	0.358	0.7	0.843	0.661	0.611	0.809	0.796	0.65	0.008	0.001	0.001	0.005	0.004	0.011	0.003	0.003	0.005
Jaccard	02_OTU_99	Species	0.261	0.336	0.727	0.868	0.652	0.618	0.828	0.84	0.669	0.004	0.002	0.001	0.004	0.003	0.005	0.003	0.002	0.004
Jaccard	02_OTU_99	OTU/ASV	0.296	0.27	0.768	0.968	0.746	0.568	0.88	0.871	0.613	0.005	0.004	0.001	0.005	0.003	0.002	0.003	0.002	0.009
Jaccard	03_ASV	Phylum	0	0.199	0.296	0.582	0.186	0.134	0.383	0.506	0.049	0.4116	0.007	0.001	0.005	0.0539	0.085	0.005	0.005	0.313
Jaccard	03_ASV	Class	0.047	0.287	0.391	0.711	0.167	0.53	0.348	0.77	0.204	0.1608	0.002	0.001	0.002	0.046	0.009	0.002	0.005	0.073
Jaccard	03_ASV	Order	0.208	0.257	0.506	0.761	0.048	0.615	0.515	0.743	0.448	0.013	0.011	0.001	0.001	0.2887	0.008	0.003	0.005	0.025
Jaccard	03_ASV	Family	0.28	0.277	0.663	0.88	0.392	0.58	0.737	0.844	0.603	0.007	0.003	0.001	0.003	0.004	0.005	0.005	0.005	0.007
Jaccard	03_ASV	Genus	0.293	0.24	0.735	0.911	0.555	0.603	0.763	0.88	0.683	0.005	0.009	0.001	0.002	0.005	0.008	0.002	0.004	0.007
Jaccard	03_ASV	Species	0.262	0.211	0.707	0.907	0.544	0.569	0.71	0.877	0.689	0.008	0.014	0.001	0.003	0.005	0.007	0.005	0.006	0.007
Jaccard	03_ASV	OTU/ASV	0.188	0.246	0.708	0.928	0.662	0.533	0.707	0.866	0.593	0.015	0.009	0.001	0.004	0.003	0.004	0.005	0.002	0.007
Bray-Curtis	01_OTU_97	Phylum	0.134	0.328	0.408	0.53	0.387	0.435	0.567	0.606	0.026	0.017	0.002	0.001	0.001	0.005	0.005	0.008	0.004	0.274
Bray-Curtis	01_OTU_97	Class	0.147	0.124	0.494	0.887	0.372	0.156	0.565	0.665	0.25	0.0569	0.050	0.001	0.003	0.025	0.045	0.001	0.008	0.055
Bray-Curtis	01_OTU_97	Order	0.13	0.156	0.536	0.863	0.432	0.235	0.537	0.661	0.339	0.0599	0.033	0.001	0.004	0.01	0.015	0.002	0.008	0.043
Bray-Curtis	01_OTU_97	Family	0.106	0.14	0.57	0.852	0.632	0.23	0.504	0.674	0.448	0.0779	0.056	0.001	0.006	0.004	0.023	0.003	0.01	0.011
Bray-Curtis	01_OTU_97	Genus	0.167	0.162	0.625	0.833	0.689	0.335	0.687	0.646	0.518	0.025	0.035	0.001	0.004	0.003	0.016	0.002	0.01	0.016
Bray-Curtis	01_OTU_97	Species	0.165	0.166	0.621	0.826	0.659	0.341	0.707	0.663	0.528	0.033	0.015	0.001	0.004	0.002	0.013	0.007	0.008	0.008
Bray-Curtis	01_OTU_97	OTU/ASV	0.183	0.188	0.67	1	0.696	0.287	0.811	0.798	0.483	0.023	0.022	0.001	0.004	0.003	0.019	0.003	0.003	0.017
Bray-Curtis	02_OTU_99	Phylum	0.137	0.33	0.413	0.533	0.398	0.439	0.568	0.615	0.028	0.015	0.001	0.001	0.005	0.007	0.008	0.007	0.002	0.290

Bray-Curtis	02_OTU_99	Class	0.146	0.121	0.493	0.885	0.365	0.15	0.572	0.667	0.256	0.048	0.060	0.001	0.004	0.029	0.051	0.005	0.004	0.069
Bray-Curtis	02_OTU_99	Order	0.128	0.154	0.534	0.867	0.426	0.23	0.541	0.667	0.332	0.0669	0.037	0.001	0.004	0.009	0.025	0.003	0.005	0.055
Bray-Curtis	02_OTU_99	Family	0.104	0.136	0.564	0.852	0.617	0.215	0.5	0.683	0.437	0.0769	0.062	0.001	0.004	0.006	0.029	0.002	0.008	0.015
Bray-Curtis	02_OTU_99	Genus	0.119	0.131	0.567	0.83	0.604	0.332	0.48	0.682	0.433	0.0559	0.056	0.001	0.004	0.005	0.026	0.002	0.006	0.014
Bray-Curtis	02_OTU_99	Species	0.181	0.167	0.652	0.985	0.613	0.318	0.724	0.772	0.482	0.026	0.025	0.001	0.004	0.002	0.017	0.003	0.002	0.018
Bray-Curtis	02_OTU_99	OTU/ASV	0.17	0.188	0.659	1	0.663	0.254	0.818	0.802	0.448	0.022	0.026	0.001	0.005	0.002	0.027	0.003	0.002	0.009
Bray-Curtis	03_ASV	Phylum	0.151	0.304	0.406	0.554	0.272	0.413	0.591	0.635	0.065	0.014	0.001	0.001	0.003	0.031	0.01	0.002	0.006	0.195
Bray-Curtis	03_ASV	Class	0.15	0.112	0.489	0.915	0.328	0.128	0.602	0.682	0.267	0.04	0.061	0.001	0.001	0.038	0.071	0.004	0.008	0.071
Bray-Curtis	03_ASV	Order	0.136	0.146	0.536	0.876	0.409	0.189	0.578	0.689	0.346	0.0539	0.044	0.001	0.002	0.017	0.035	0.001	0.003	0.056
Bray-Curtis	03_ASV	Family	0.111	0.131	0.577	0.863	0.63	0.18	0.53	0.733	0.454	0.0709	0.058	0.001	0.002	0.003	0.025	0.003	0.005	0.017
Bray-Curtis	03_ASV	Genus	0.128	0.126	0.576	0.843	0.593	0.294	0.517	0.722	0.441	0.0539	0.041	0.001	0.003	0.003	0.019	0.005	0.005	0.017
Bray-Curtis	03_ASV	Species	0.188	0.159	0.656	0.994	0.607	0.268	0.746	0.806	0.485	0.028	0.03	0.001	0.004	0.001	0.023	0.005	0.006	0.01
Bray-Curtis	03_ASV	OTU/ASV	0.184	0.191	0.665	1	0.668	0.244	0.846	0.809	0.474	0.015	0.02	0.001	0.002	0.006	0.025	0.004	0.007	0.018
